# Uncovering Intrinsic Modular Organization of Spontaneous Brain Activity in Humans

**DOI:** 10.1371/journal.pone.0005226

**Published:** 2009-04-21

**Authors:** Yong He, Jinhui Wang, Liang Wang, Zhang J. Chen, Chaogan Yan, Hong Yang, Hehan Tang, Chaozhe Zhu, Qiyong Gong, Yufeng Zang, Alan C. Evans

**Affiliations:** 1 State Key Laboratory of Cognitive Neuroscience, Beijing Normal University, Beijing, China; 2 McConnell Brain Imaging Centre, Montreal Neurological Institute, McGill University, Montreal, Quebec, Canada; 3 Department of Radiology, First Affiliated Hospital of College of Medical Science, Zhejiang University, Hangzhou, China; 4 Huaxi MR Research Center, Department of Radiology, West China Hospital of Sichuan University, Chengdu, China; Indiana University, United States of America

## Abstract

The characterization of topological architecture of complex brain networks is one of the most challenging issues in neuroscience. Slow (<0.1 Hz), spontaneous fluctuations of the blood oxygen level dependent (BOLD) signal in functional magnetic resonance imaging are thought to be potentially important for the reflection of spontaneous neuronal activity. Many studies have shown that these fluctuations are highly coherent within anatomically or functionally linked areas of the brain. However, the underlying topological mechanisms responsible for these coherent intrinsic or spontaneous fluctuations are still poorly understood. Here, we apply modern network analysis techniques to investigate how spontaneous neuronal activities in the human brain derived from the resting-state BOLD signals are topologically organized at both the temporal and spatial scales. We first show that the spontaneous brain functional networks have an intrinsically cohesive modular structure in which the connections between regions are much denser within modules than between them. These identified modules are found to be closely associated with several well known functionally interconnected subsystems such as the somatosensory/motor, auditory, attention, visual, subcortical, and the “default” system. Specifically, we demonstrate that the module-specific topological features can not be captured by means of computing the corresponding global network parameters, suggesting a unique organization within each module. Finally, we identify several pivotal network connectors and paths (predominantly associated with the association and limbic/paralimbic cortex regions) that are vital for the global coordination of information flow over the whole network, and we find that their lesions (deletions) critically affect the stability and robustness of the brain functional system. Together, our results demonstrate the highly organized modular architecture and associated topological properties in the temporal and spatial brain functional networks of the human brain that underlie spontaneous neuronal dynamics, which provides important implications for our understanding of how intrinsically coherent spontaneous brain activity has evolved into an optimal neuronal architecture to support global computation and information integration in the absence of specific stimuli or behaviors.

## Introduction

Spontaneous neuronal activity refers to the brain activity that is intrinsically generated in the absence of explicit inputs or outputs [1]. For the brain functional studies, the investigation of the intrinsic or spontaneous brain activity is thought to be vital since it is able to (i) represent unconstrained conscious mental activity, (ii) facilitate responses to tasks or stimuli, and (iii) assess brain-behavior relationship [for reviews, see 1], [2], [3].

Recently, many researchers have focused on exploring the nature of the brain's intrinsic functional activity by examining the slow (<0.1 Hz), spontaneous blood oxygen level dependent (BOLD) fluctuations observed in the resting state using functional magnetic resonance imaging (fMRI). For instance, several researchers have exclusively studied regional characteristics of spontaneous BOLD signals, such as regional neuronal coherence [4], [5] and fractal complexity [6]. Alternatively, several functional connectivity fMRI studies [7], [8] have examined correlations in the spontaneous BOLD fluctuations among different brain regions and demonstrated that many neuroanatomical systems tend to be highly coherent in their spontaneous activity, including the motor [8]–[10], auditory [11], visual [10], language [12], default-mode [13], [14] and attention systems [15]. Some of these functional systems have also been identified using multivariate statistical approaches such as hierarchical clustering [16] and independent component analysis (ICA) [17]–[20]. With the recent advent of modern network analysis based on graph theory [21], [22], several studies have investigated the large-scale topological organization of these coherent spontaneous brain activities, and revealed many important statistical characteristics underlying the functional organization of the human brain, including the small-world property [16], [23]–[25], high efficiency at a low wiring cost [24]–[27], and truncated power-law degree distribution [23], [24], [27]. These global network properties have been shown to be largely compatible with those observed in the human brain structural networks [28]–[32]. Here, we will focus on an important question concerning the network modularity of the ongoing, spontaneous BOLD activity in the human brain.

Modularity, presumably shaped by evolutionary constraints, is thought to be one of the main organizing principles of most complex systems, including social, economical and biological networks [33]–[35], [for a review, see 36]. Detection and characterization of modular structure in the brain system can help us to identify groups of anatomically and/or functionally associated components that perform specific biological functions. Several recent studies have attempted to investigate various aspects of modular organization of large-scale *structural* brain networks in both mammalians and humans. By analyzing anatomical connectivity data in the cat cerebral cortex [37], Zhou and colleagues have demonstrated that there exist structurally interconnected modules in the cat brain network, which broadly agree with several well-defined functional subdivisions such as the somatosensory-motor, auditory, visual, and fronto-limbic [38]. In the human cerebral cortex, the modular architecture of structural connectivity patterns has been also demonstrated by using the cortical thickness measurement from structural MRI [39] and white matter tracts from diffusion spectrum imaging [40], respectively. There are also a few recent studies reporting *functional* modular organization of spontaneous neuronal activity in the brain networks using spontaneous BOLD fluctuations derived from resting-state fMRI data in the rats [41] and healthy human subjects [42], [43] (we will discuss the similarities and differences among these studies in the Discussion section).

In the present study, we performed a comprehensive modularity analysis of human brain functional networks by examining both temporal and spatial correlation patterns of spontaneous BOLD fluctuations derived from resting-state fMRI. Temporal correlation patterns were obtained by measuring the extent of similarity of BOLD time series between regional pairs, but spatial correlation patterns were obtained by measuring the extent of similarity of temporal correlation maps of BOLD signals between regional pairs (also see Materials and Methods). To address our issues, we first constructed the large-scale human brain functional networks at both the temporal and spatial scales, and then revealed their intrinsically modular architectures that underlie spontaneous neuronal dynamics. We further computed the topological parameters for each module, and determined whether these module-specific properties could also be characterized by the corresponding global network parameters. Finally, we identified the pivotal brain regions and connections of the spontaneous brain functional networks that are crucial in controlling the information flow of the whole networks, and evaluated how their lesions (deletions) would affect the topological stability and robustness of the brain functional networks.

## Results

### Construction of the Temporal and Spatial Brain Functional Networks

**Figure 1:**
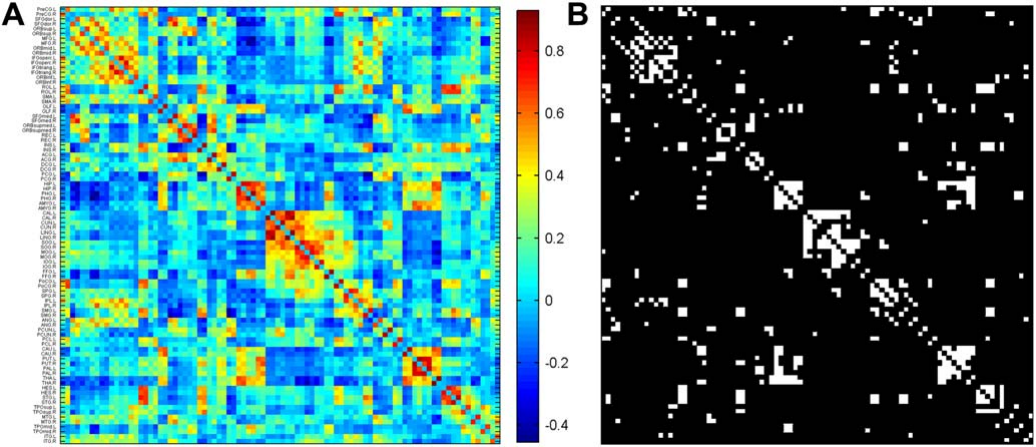
Inter-regional correlation matrix and its functional connectivity backbone. (A) The mean correlation matrix is obtained by averaging a set of correlation matrices across subjects where individual correlation matrix is acquired by calculating Pearson correlation coefficients of time series between every pair of brain regions. The color bar indicates the correlation coefficients. The black arrow in the color bar indicates the threshold value (r = 0.44) that was used to obtain the binarized matrix (B). For the abbreviations of the regions, see Table S1. (B) The functional connectivity backbone (binarized matrix) is obtained by thresholding the mean correlation matrix using a Bonforroni-corrected procedure (*P*<0.001). Significant correlations between regions are marked in white squares and black squares otherwise. Notably, the binarized matrix describes the basic topological organization of the spontaneous human brain functional network.

In the current study, we employed resting-state BOLD fMRI signal to construct spontaneous brain functional networks at both the temporal and spatial scales. First, a prior brain atlas [44] was utilized to parcellate the whole brain into ninety cortical and subcortical regions (Table S1), with each of them representing a single node in the brain functional networks. We then acquired individual temporal correlation matrices of the ninety brain regions by computing the correlation coefficients between the time-courses of every pair of regions (Figure 1A). A random-effect one-sample *t* test was further performed on these correlation matrices in an element-by-element manner to obtain the significance level (i.e. *P* value) of each inter-regional correlation across the subjects. Finally, the *P*-value matrix was thresholded by using a conservative Bonferroni-corrected *P* value (*P* = 0.001) to reduce the chance of false positives, which resulted in a binarized matrix (sparsity = 8.41%) that captured the functional connectivity backbone underlying the topological organization of spontaneous human brain activity at a time domain (Figure 1B). Unless stated otherwise, we will mainly report our results using this threshold. However, considering that different thresholds would have an effect on the number of links in the resulting brain networks, we also evaluated the topological stability of the brain functional networks by applying multiple statistical thresholds (Bonferroni-corrected *P* values of 0.005, 0.01, 0.05 and 0.10 which correspond to a network sparsity of 10.79%, 12.16%, 15.38% and 16.78%, respectively) to the *P*-value matrix. In addition to the abovementioned temporal brain functional networks, in this study, we also constructed spatial brain functional networks derived from thresholding inter-regional spatial correlation matrices that were composed of correlation coefficients between every pair of vectors in the temporal correlation matrices above (see Materials and Methods). The analysis of the spatial brain functional networks was similar to that of the temporal brain functional networks.

### Modularity of the Functional Brain Networks

**Figure 2:**
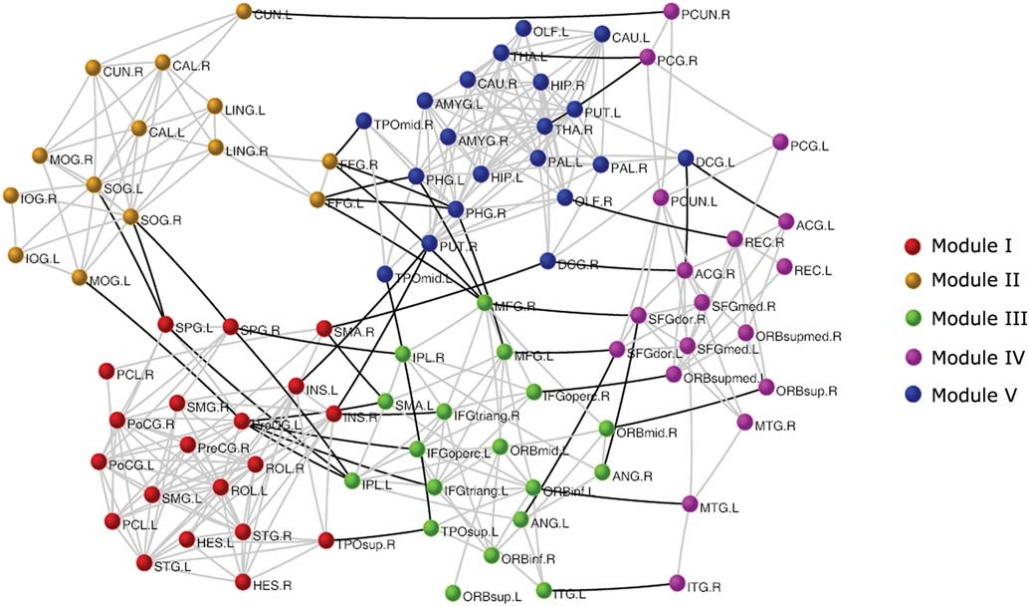
The modular architecture of the human brain functional network. We identify five functional modules in the spontaneous brain functional network represented by five different colors. The geometric distance between two brain regions on the drawing space approximates the shortest path length between them. The network is visualized with the Pajek software package (http://vlado.fmf.uni-lj.si/pub/networks/pajek/) using a Kamada-Kawai layout algorithm. The intra-module and inter-module connections are shown in gray and dark lines, respectively. For the abbreviations of the regions, see Table S1.

**Figure 3:**
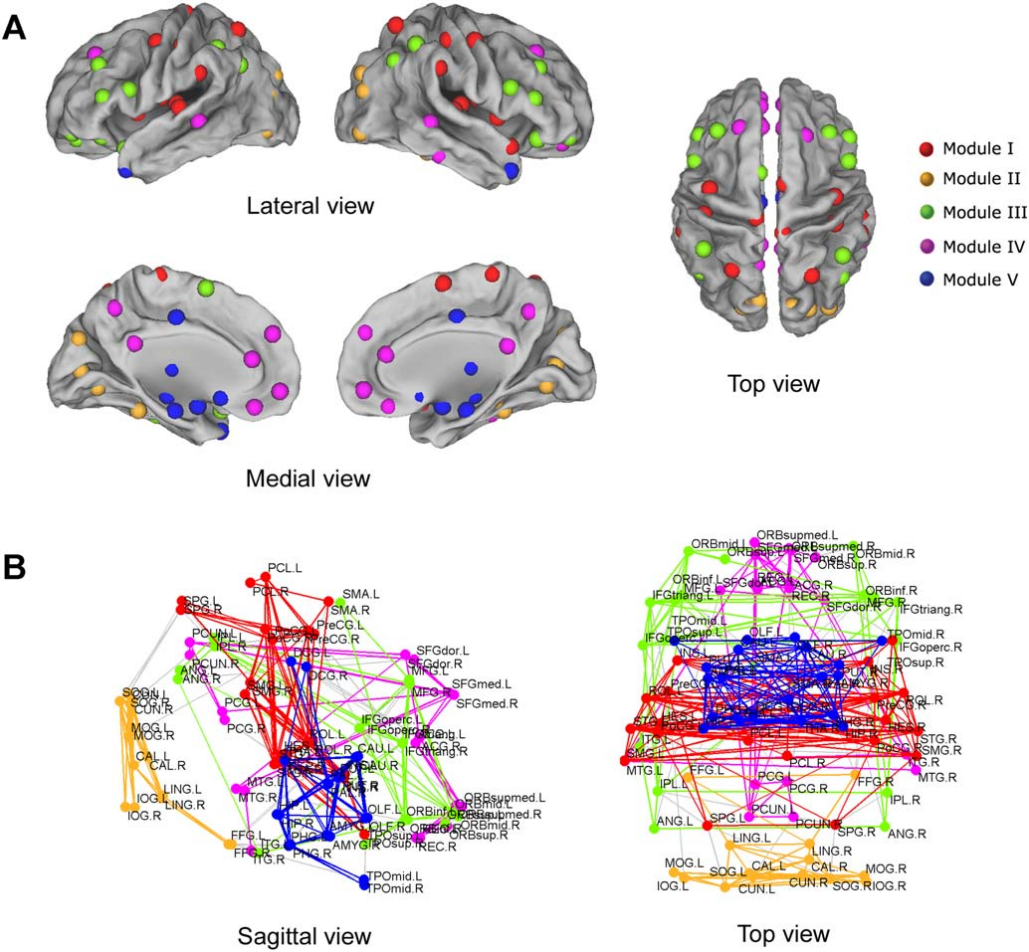
Surface and anatomical representation of modular architecture of the human brain functional network. (A) All of 90 brain regions are marked by using different colored spheres (different colors represent distinct network modules) and further mapped onto the cortical surfaces at the lateral, medial and top views, respectively, by using the Caret software [84]. Notably, the regions are located according to their centroid stereotaxic coordinates. For the visualization purpose, the subcortical regions are projected to the medial cortical surface according to their y and z centroid stereotaxic coordinates. (B) Sagittal and top views of the spontaneous brain functional network. The nodes and edges within each module are marked in one single color. The inter-module connections are shown in gray lines. For the abbreviations of the regions, see Table S1.

**Figure 4:**
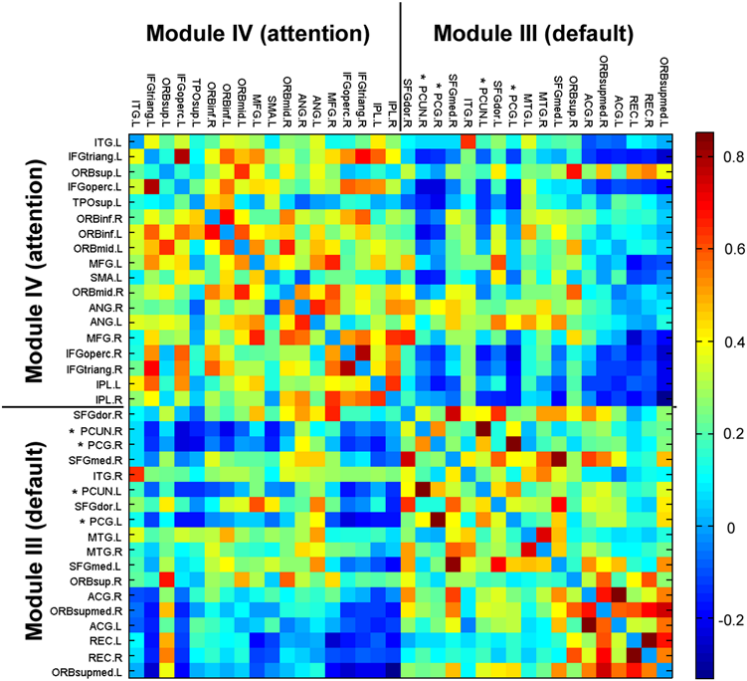
The anti-correlation map between module III and module IV. We show the inter-regional correlations between module III and module IV. The two key regions in the ‘default’ subnetwork (module III), the posterior cingulate cortex and precuneus (asterisk signs), exhibit dramatically negative correlations with most of brain regions in the attention subnetwork (module IV). The color bar indicates the correlation coefficients. Note that the correlation matrix is extracted from Figure 1A.

A network module is referred as a set of nodes with denser links among them, but sparser with the rest of the network. It has been shown that modularity is one of the most fundamental and intriguing properties of many biological networks [36]. To determine whether the spontaneous brain functional networks also have a modular structure at the temporal scale, we employed a simulated annealing approach [45], [46] to find the network partitions that maximize the modularity (see Materials and Methods). Notably, the modular detection process did not take into account of prior knowledge regarding the functionality of any brain regions. As a result, a maximum modularity (Q_max_ = 0.66, Z-score = 45.25) was reached when the brain functional network was separated into 5 modules (I, II, III, IV, V in Figure 2 and Figure 3). Module I included 20 regions mostly from (pre)motor, parietal and temporal cortices such as right supplementary motor area, bilateral precentral gyrus, postcentral gyrus, paracentral lobule, superior parietal gyrus, supramarginal gyrus, insula, superior temporal gyrus, and heschl gyrus that are mainly associated with the somatosensory, motor and auditory functions [47]. The result was consistent with several recent resting-state fMRI studies using ICA demonstrating that these motor- and auditory-related areas were located at one single component [20]. The result was also compatible with a recent graph theoretical analysis of human brain functional network in which Meunier et al. [43] used resting-state fMRI measurement to identify a central module that was mainly composed of the motor and auditory areas. Module II included all of 14 regions from the occipital lobe, namely bilateral superior, middle and inferior occipital gyrus, cuneus, calcarine fissure, fusiform gyrus and lingual gyrus that are primarily specialized for visual processing. This result was consistent with many previous resting-state fMRI studies [16]–[20], [43]. The 18-region module III was mainly composed of regions from lateral frontal and parietal cortices such as bilateral middle frontal gyrus, inferior frontal gyrus (both opercular and triangular part), angular gyrus, and inferior parietal lobe that are known to be predominantly involved in attention processing [48]. This finding was also in agreement with many previous studies showing coherent spontaneous BOLD fluctuations in the front-parietal system [15], [18]–[20].The other 18-region module IV consisted of regions mostly from medial frontal and parietal cortices, and lateral temporal cortex such as bilateral anterior cingulate gyrus, medial superior frontal gyrus, posterior cingulate gyrus, precuneus and middle temporal gyrus that are the key components of the ‘default’ network as described by Raichle et al. [49] and Greicius et al. [14]. The last 20-member module V included regions such as bilateral parahippocampal gyrus, hippocampus, amygdale, temporal pole, olfactory cortex, thalamus, caudate, putamen and pallidum that are components of limbic/paralimbic and subcortical systems. Interestingly, we found that several major ‘default’ regions in module IV (e.g. posterior cingulate cortex and precuneus) showed strongly negative correlations with most regions in module III that are associated with attention function (Figure 4), which was in accordance with recent findings of anti-correlations between the default and attention subsystems [13], [50]. It was noteworthy that the significant modular architecture shown here was also reproduced in both the temporal and spatial brain functional networks constructed using distinct statistical thresholds (Figure S2 and Table S2). Interestingly, when the modularity detection algorithm was further applied to individual modules, it was able to identify several sub-modules (Figure S1). For instance, module I was subdivided two small modules (Q_max_ = 0.20, Z-score = 7.01) that corresponded to the somatosensory/motor and auditory systems, respectively, which was consistent with previous studies showing that the regions were associated with different functional components or clusters [16], [18], [39]. Module IV was subdivided into three small modules (Q_max_ = 0.42, Z-score = 5.81) that approximately corresponded to the anterior, middle, and posterior parts of the ‘default’ network, which was also compatible with previous studies of functional subdivisions in the brain system [51]. On the basis of the recursive analysis of modular detection algorithm as well as previous studies [42], we speculate that the spontaneous functional networks of the human brain are likely to be topologically organized into a hierarchical modular structure at a macroscale (i.e. region level).

### Global versus Module-Specific Average Network Properties

We have identified a markedly modular structure in both the temporal and spatial spontaneous brain functional networks in humans as shown previously. Several recent studies have demonstrated that the large-scale brain networks have a small-world topology at a global level [16], [23]–[31], [52], characterized by high clustering and short path length [53], or high efficiency at a low wiring cost [54]. A key question we further posed here is whether the local topological properties within each module can be characterized by means of these global network parameters, such as the average degree (*<k>*), the shortest path length (*L_p_*), the clustering coefficient (*C_p_*), the local (*E_loc_*) and global efficiency (*E_glob_*). To address this issue, we compared the local network parameters within each module with the corresponding global network parameters obtained by a randomization procedure [55] (see Materials and Methods). The analysis of the global brain networks exhibited a small-world-like topology (Table S3, S4) as expected. However, more importantly, we found that, except for the clustering coefficient, almost all average network properties of each module significantly (*P*<0.05) differ from the corresponding global average of the whole brain networks (Table 1 and Table S5). Likewise, the analysis of spatial brain functional networks demonstrated similar results (Table S6, S7). These findings strongly implicate that the average properties of the global brain functional networks can not be representative of individual module-specific properties because each module in the network contains a specific topological structure.

**Table 1:** Global vs. module-specific properties in the human brain functional networks.

Threshold, S	*r_<k>_*	*r_Cp_*	*r_Lp_*	*r_Eloc_*	*r_Eglob_*
8.41%	1.00 (0.00)	0.80 (0.00)	1.00 (0.00)	1.00 (0.00)	1.00 (0.00)
10.79%	1.00 (0.00)	1.00 (0.00)	1.00 (0.00)	1.00 (0.00)	1.00 (0.00)
12.16%	1.00 (0.00)	1.00 (0.00)	1.00 (0.00)	1.00 (0.00)	1.00 (0.00)
15.38%	1.00 (0.00)	0.81 (0.05)	1.00 (0.00)	1.00 (0.00)	1.00 (0.00)
16.78%	1.00 (0.00)	1.00 (0.00)	1.00 (0.00)	1.00 (0.00)	1.00 (0.00)

The table illustrates the fraction *r* of modules (and standard deviation) whose topological parameters significantly (*P*<0.05) differ from the corresponding global network parameters. *<k>*, average degree; *C_p_*, clustering coefficient; *L_p_*, characteristic path length; *E_glob_*, global efficiency; and *E_loc_*, local efficiency. Notably, the first column (network threshold, *S*) denotes the network sparsity thresholds corresponding to the Bonferroni-corrected significance levels (*P* = 0.001, 0.005, 0.01, 0.05 and 0.10, respectively) that were used to construct brain functional networks at the temporal scale. Under each threshold, there were 5 modules that were identified in the temporal functional brain networks by using the modular identification algorithms (Table S2). For details, see Materials and Methods.

### Node Diversity of the Functional Brain Networks

#### Roles of nodes

**Figure 5:**
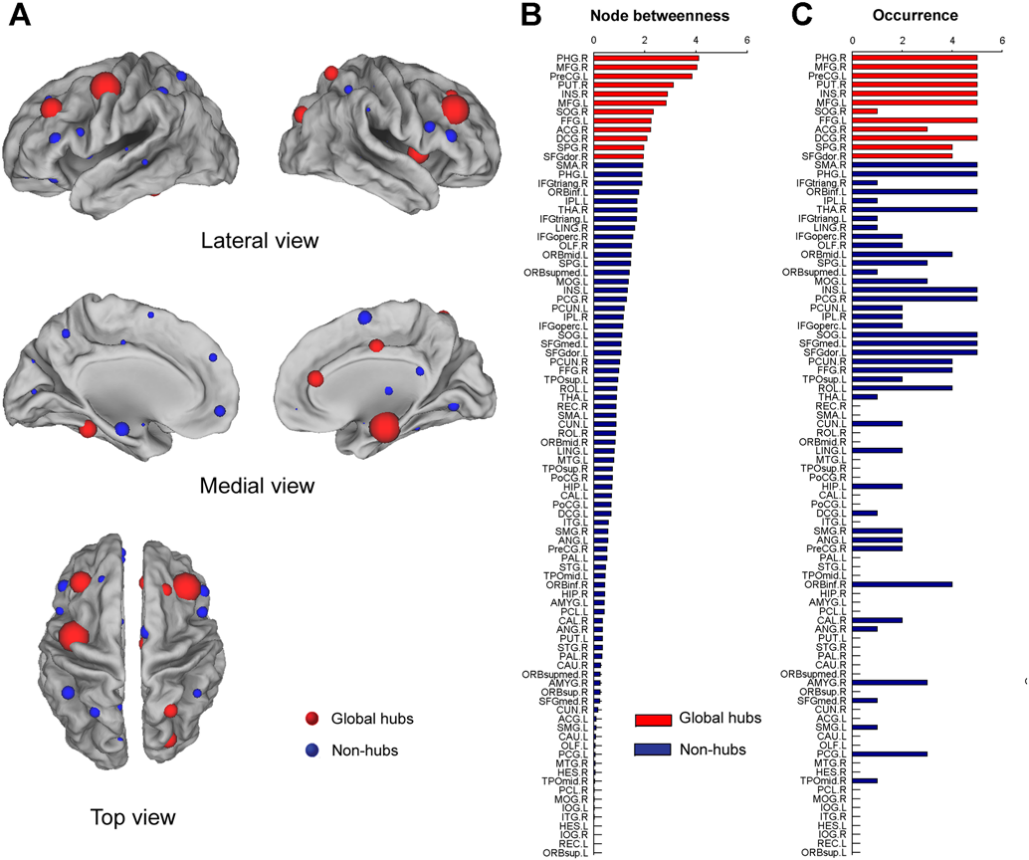
The global hubs with high topological centralities in the human brain functional networks. (A) The surface visualization of all 90 brain regions with node sizes indicating their relative node betweenness centrality, *N_bc_* values. Regions with *N_bc_*>mean+std are considered as hubs (red colors) and non-hubs (blue colors) otherwise. (Figure 1B). (B) The bar plot of all 90 brain regions in a descending order of their relative node betweenness centrality. Red and blue color bars indicate hub regions and non-hub regions in the brain network, respectively. For the abbreviations of the regions, see Table S1. (C) The bar plot of the occurrence that brain regions show high *N_bc_* values (>mean) in the functional brain networks constructed at all selected statistical thresholds (*P* = 0.001, 0.005, 0.01, 0.05 and 0.10). If one region shows a high occurrence, it indicates that this region has a high topological centrality in the spontaneous brain functional networks and is insensitive to the selection of statistical thresholds.

**Figure 6:**
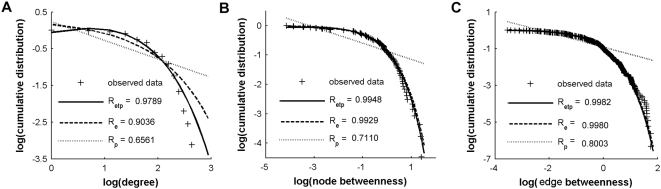
Topological distribution of the human brain functional networks. (A) Log-log plot of the cumulative probability of node degree distribution. (B) Log-log plot of the cumulative probability of relative node betweenness distribution. (C) Log-log plot of the cumulative probability of relative edge betweenness distribution. The solid, dashed and dotted lines indicate the fits of exponentially truncated power law [*p(x)∼x^α−1^e^x/xc^*], exponential [*p(x)∼e^x/xc^*], and power law [*p(x)∼x^α−1^*], respectively. R-squared values indicate the goodness of the fits. R_etp_, R-squared value for an exponentially truncated power law fit; R_e_, R-squared value for an exponential fit; and R_p_, R-squared value for a power law fit.

We first investigated the global role of every node (i.e. region) in the brain networks by examining their relative betweenness centrality [56], *N_bc_* (see Materials and Methods). The higher *N_bc_* for a region, the more important the region is to the whole network. Twelve regions including 6 heteromodal or unimodal association cortex regions, 4 limbic/paralimbic cortex regions, 1 primary motor cortex region and 1 subcortical region were identified as the global hubs (*N_bc_*>mean+std) (Table 2, Figure 5A and 5B). Notably, these identified hub regions were predominately located at those recently evolved association cortex regions [middle frontal gyrus, superior occipital gyrus, fusiform gyrus, superior parietal gyrus and superior frontal gyrus (dorsolateral)] and primitive paralimbic/limbic cortex regions (parahippocampal gyrus, insula, anterior and middle cingulate gyrus) [47], most of which have been recently found to tend to have high regional efficiency or centrality in the functional [23] and structural [28], [39] brain networks in humans. These global hubs were also obtained in the brain networks derived from using different statistical thresholds at the temporal (Figure 5C) and spatial scales (Figure S3). Furthermore, we found that, in the spontaneous brain functional networks, both the node betweenness and degree distribution followed an exponentially truncated power law distribution pattern as opposed to a scale-free distribution (Figure 6 and Figure S4), implying that the lack of nodes with extremely high centrality.

**Table 2:** The global hubs of the human brain functional network.

Region	Class	*N_bc_(i)*	*K(i)*	*C(i)*	*L(i)*	Module	Role
PHG.R	Paralimbic	4.11	14	0.34	2.22	V	R1
MFG.R	Association	4.04	10	0.16	2.22	III	R3
PreCG.L	Primary	3.85	11	0.29	2.32	I	R3
PUT.R	Subcortical	3.12	10	0.42	2.46	V	R3
INS.R	Paralimbic	2.89	11	0.56	2.31	I	R3
MFG.L	Association	2.83	8	0.29	2.33	III	R3
SOG.R	Association	2.33	12	0.39	2.59	II	R2
FFG.L	Association	2.25	6	0.40	2.57	II	R3
ACG.R	Paralimbic	2.22	8	0.29	2.61	IV	R1
DCG.R	Paralimbic	2.08	6	0.27	2.55	V	R3
SPG.R	Association	1.96	7	0.33	2.48	I	R3
SFGdor.R	Association	1.94	8	0.36	2.53	IV	R3

The hub regions (*N_bc_(i)*>mean+SD) are listed in a decreasing order of their relative node betweenness centrality and further classified into association, primary, limbic/paralimbic and subcortical regions as described by Mesulam (2000). *N_bc_(i)*, *K(i)*, *C(i)* and *L(i)* denote the relative node betweenness, degree, clustering coefficient and shortest path length of node *i*, respectively. The Module column denotes the functional modules that the hub regions belong to, and the Role column denotes the roles that the hub regions play in terms of their intra- and inter-module connectivity patterns (See Materials and Methods). R: right; L: left. For the description of the abbreviations, see Table S1.

**Figure 7:**
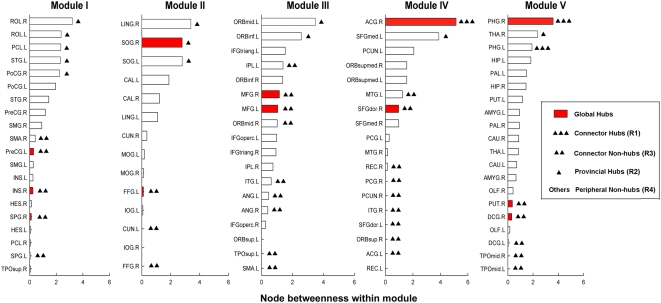
The classifications of brain regions by means of their intra- and inter-module connectivity patterns. All of 90 brain regions are divided into four categories in terms of their relative regional within-module betweenness centrality (*N_bc_^s^*) and participant coefficient (*PC*) (see Materials and Methods). The bars denote the ranked *N_bc_^s^* values within modules. The identified global hubs in the brain functional networks are marked in red colors and are found to be mainly composed of the network connectors. For the abbreviations of the regions, see Table S1.

**Figure 8:**
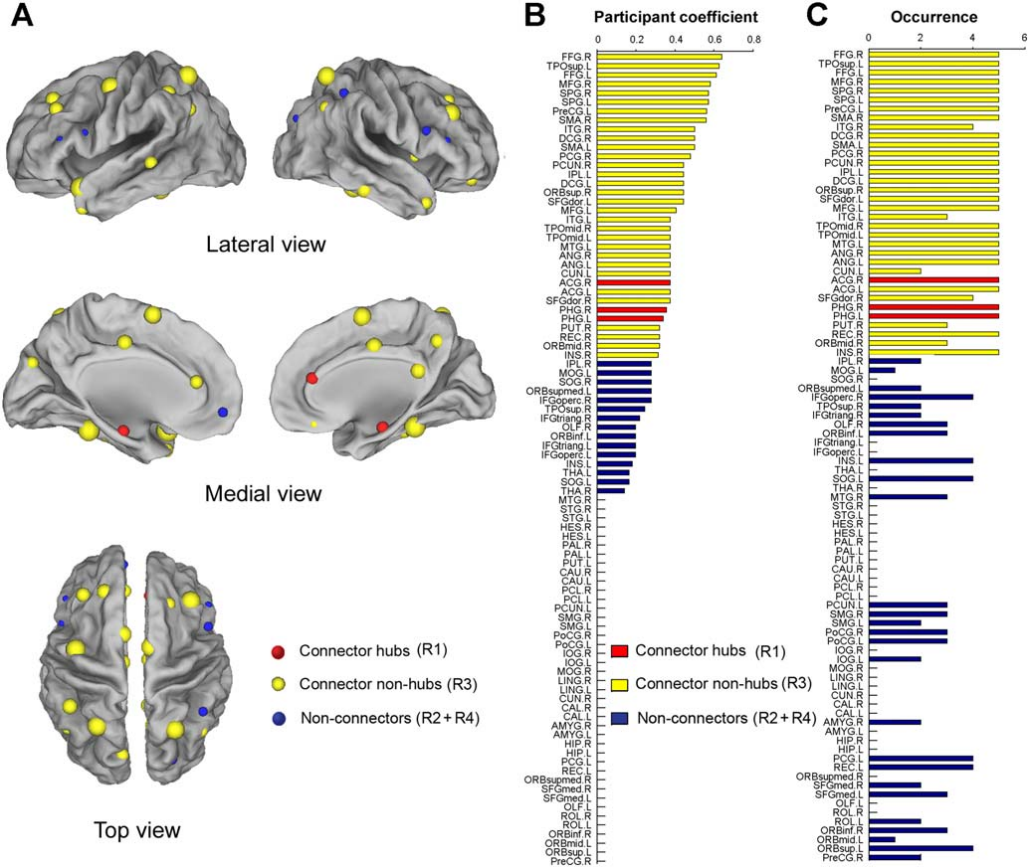
Surface representation of the network connectors. (A) The surface visualization of all 90 brain regions with node sizes indicating their participant coefficient (*PC*) values. Regions with *PC*>0.30 are considered connectors (red and yellow colors) and otherwise non-connectors (blue colors). (B) The bar plot of all 90 brain regions in a descending order of their *PC* values. Red, yellow and blue color bars indicate the connector hubs, connector non-hubs, and non-connectors in the brain network, respectively. For the abbreviations of the regions, see Table S1. (C) The bar plot of the occurrence that brain regions show high *PC* values (>0.30) in the functional brain networks constructed at all selected statistical thresholds (*P* = 0.001, 0.005, 0.01, 0.05 and 0.10). If one region shows a high occurrence, it indicates that this region has a high participant coefficient in the spontaneous brain functional networks and is insensitive to the selection of statistical thresholds.

In this study, we further characterized the role of each node in the brain functional networks according to their patterns of intra- and inter-module connections. Two measurements were used: the relative within-module betweenness centrality (*N_bc_^s^*), which quantifies the level of control a region has over the information flow among others within the same module, and participant coefficient (*PC*), which quantifies the extent of a region's connections to distinct functional modules [33], [34]. Based on the two measures, we divided all of network nodes into four categories (see Materials and Methods): connector hubs (R1, 3), provincial hubs (R2, 12), connector non-hubs (R3, 31), and peripheral non-hubs (R4, 44) (Figure 7, Figure 8A and 8B). Importantly, we found that 11 out of the 12 global hubs identified previously belonged to either R1 or R3 connectors that had many inter-module connections (Figure 7), thus constituting a functional core that played a critical role in the coordination of information flow over the whole network. Further analysis indicated that these identified connectors were also consistent with in the brain networks derived from different statistical thresholds at both the temporal (Figure 8C) and spatial scales (Figure S5). Additionally, we also noted that there were a minimum amount of overlap between the connectors and within-module hubs (Figure 7), suggesting that the brain regions in the spontaneous brain networks are likely to be responsible for distinct aspects of intra- and inter-module communications.

#### Node removal

**Figure 9:**
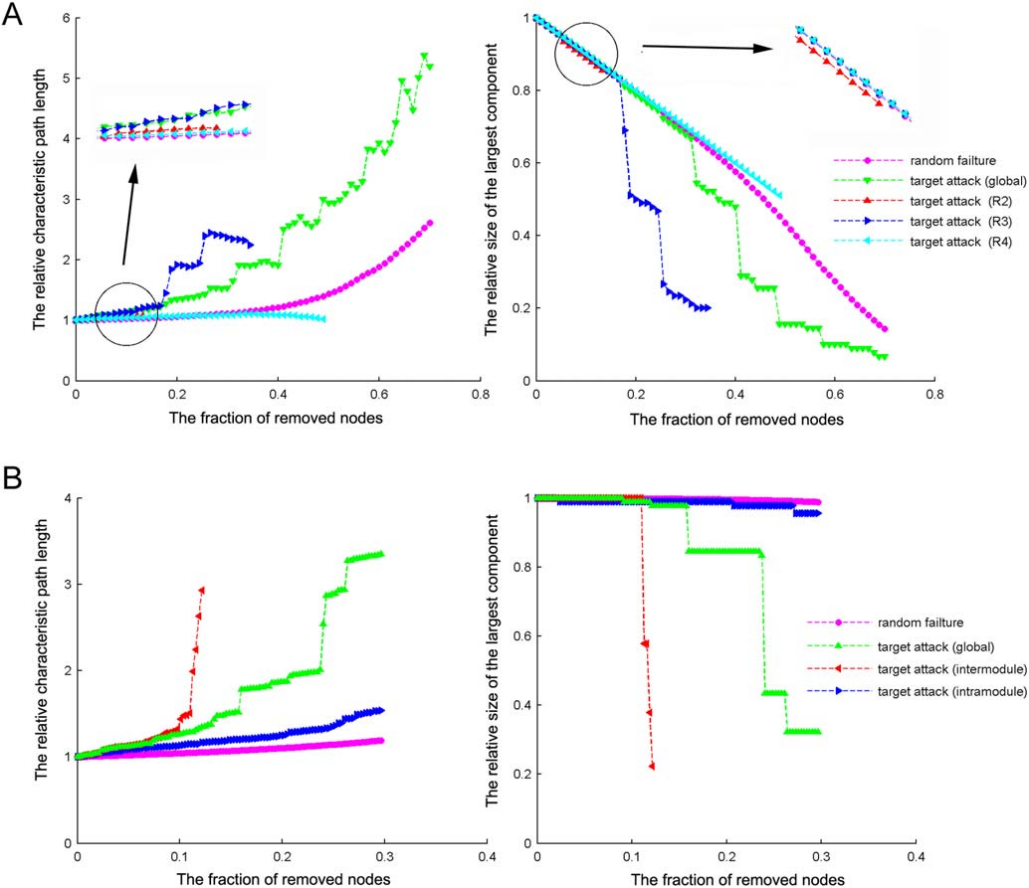
Topological robustness of the human brain functional network. (A) Network robustness in response to node (brain regions) lesions. The graphs show the changes in the relative characteristic path length (left) and the size of the largest connected component (right) as a function of the fraction of removed nodes. The removal of R1 connector hubs is omitted because only 3 nodes are included in the brain networks. (B) Network robustness in response to edge (connections) lesions. The graphs show the changes in the relative characteristic path length (left) and the size of the largest connected component (right) as a function of the fraction of removed edges.

To assess the effect of nodal ‘lesions’ on the overall topology of brain functional networks, in the present investigation we performed a simulation analysis [23], [29], [57], [58] to examine the network performance after individual nodes were continuously removed in a manner of random failure or targeted attack, respectively (see Materials and Methods). As expected, the continuous attacks on the global hubs (i.e. regions with high *N_bc_*) had a more dramatic effect on the brain functional network performance than the random failure of regions (Figure 9A). However, further analysis revealed striking differences in the removal of the nodes with different roles in terms of their intra- and inter-module communications. Attacks against R3 connectors had a significantly more deleterious effect on the network integrity as compared to the removal of R2 provincial hubs and R4 peripheral non-hubs that resembled random failures (Figure 9A). Attacks on R1 connector hubs were omitted since only 3 regions were obtained here. Interestingly, we observed that the network integrity was sharply decreased with a removal of a certain percentage of nodes [e.g. 15 R3 connectors (15/90; 16.67%) in Figure 9A]. This phenomenon implies that there may be a critical point in the level of the brain network tolerance in which the system would collapse when attacked. Together, our results indicate that the connector regions linking different functional modules are more responsible in keeping the robustness and stability of the brain functional networks.

### Edge Diversity of the Functional Brain Networks

#### Roles of edges

In this study, we also characterized the role of each edge (i.e. functional connection between two regions) in the brain functional network in terms of its relative edge betweenness centrality [56], *E_bc_* (see Materials and Methods). Table 3 showed the most pivotal 42 edges (i.e. bridges) that were ranked according to their *E_bc_*. In the brain network, the proportion of edges with high *E_bc_* was found to be relatively rare (42/337; 12.46%) as indicated by a truncated power-law edge betweenness distribution (Figure 6, Figure S4). Moreover, we found that these bridges were mainly composed of inter-module connections (25/42; 59.52%), despite the fact that most network connections were intra-module (296/337; 87.83%). Further statistical analysis revealed that, of these edges with high *E_bc_*, the number of inter-module connections was significantly (chi-square test, *χ^2^*(1) = 104.07; *P* = 0.000) more than that of intra-module connections. Moreover, we noted that these edges were mainly associated with those global hubs with high *N_bc_* (28/42; 66.67%) or inter-module R1/R3 connectors with high *PC* (40/42; 95.24%) shown in the previous section. The bridge edges were also found to be composed of 11 inter-hemispheric, 14 inter-lobe and 17 intra-lobe connections (Table 3), which were approximately consistent with our recent studies in the structural brain networks [30], [39]. Additionally, we also observed that there existed a few number of edges with high *E_bc_*, but linking two R4 peripheral non-hub nodes such as the edge between the inferior frontal gyrus (opercular) and superior frontal gyrus (medial orbital) (Table 3).

**Table 3:** The “bridge” connections of the human brain functional network.

Region A	Region B	Class	*E_bc_*({i,j})	Module	Role
MFG.L	PHG.R	Inter-H	5.74	III-V	R3-R1
SMA.R	DCG.R	Inter-L	5.18	I-V	R3-R3
PreCG.L	MOG.L	Inter-L	4.99	I-II	R3-R4
INS.R	PUT.R	Inter-L	4.90	I-V	R3-R3
INS.R	IFGtriang.R	Inter-L	4.87	I-III	R3-R4
MFG.R	SFGdor.R	Intra-L	4.55	III-IV	R3-R3
IFGoperc.R	ORBsupmed.L	Inter-H	4.18	III-IV	R4-R4
INS.L	PUT.R	Inter-H	4.16	I-V	R4-R3
SPG.R	SOG.R	Inter-L	3.93	I-II	R3-R2
MFG.R	PHG.L	Inter-H	3.55	III-V	R3-R1
REC.R	OLF.R	Intra-L	3.45	IV-V	R3-R4
LING.L	FFG.L	Intra-L	3.39	II-II	R4-R3
CUN.L	PCUN.R	Inter-H	3.29	II-IV	R3-R3
ACG.R	DCG.R	Intra-L	3.24	IV-V	R1-R3
PreCG.L	IFGtriang.L	Intra-L	3.21	I-III	R3-R4
FFG.L	PHG.R	Inter-H	3.08	II-V	R3-R1
SPG.R	IPL.R	Intra-L	2.92	I-III	R3-R4
ORBinf.L	MTG.L	Inter-L	2.91	III-IV	R2-R3
LING.R	FFG.L	Inter-H	2.84	II-II	R2-R3
FFG.L	MFG.R	Inter-H	2.83	II-III	R3-R3
MFG.L	SFGdor.L	Intra-L	2.80	III-IV	R3-R3
PreCG.L	IFGoperc.L	Intra-L	2.75	I-III	R3-R4
MFG.R	IFGtriang.R	Intra-L	2.73	III-III	R3-R4
PCG.R	THA.R	Inter-L	2.72	IV-V	R3-R2
LING.R	FFG.R	Intra-L	2.69	II-II	R2-R3
TPOsup.R	TPOsup.L	Inter-H	2.51	I-III	R4-R3
DCG.R	THA.R	Inter-L	2.50	V-V	R3-R2
PreCG.L	ROL.L	Intra-L	2.48	I-I	R3-R2
PCUN.L	PCUN.R	Inter-H	2.35	IV-IV	R4-R3
SPG.L	SOG.L	Inter-L	2.30	I-II	R3-R2
ORBsup.L	ORBmid.L	Intra-L	2.26	III-III	R4-R2
SFGmed.L	ACG.R	Inter-H	2.21	IV-IV	R2-R1
SPG.R	SMG.R	Intra-L	2.15	I-I	R3-R4
ROL.R	SMA.R	Intra-L	2.14	I-I	R2-R3
IPL.L	ANG.L	Intra-L	2.13	III-III	R3-R3
IFGtriang.L	ITG.L	Inter-L	2.12	III-III	R4-R3
SFGdor.R	ACG.R	Inter-L	2.10	IV-IV	R3-R1
OLF.R	PHG.R	Inter-L	2.07	V-V	R4-R1
FFG.R	MFG.R	Intra-L	2.06	II-III	R3-R3
SPG.L	IPL.L	Intra-L	2.03	I-III	R3-R3
PCG.R	PCUN.R	Inter-L	2.00	IV-IV	R3-R3
OLF.R	PUT.R	Inter-L	2.00	V-V	R4-R3

The “bridge” connections (*E_bc_*({i,j})>mean+SD) are listed in a descending order of their relative edge betweenness centrality and further classified into Inter-H (inter-hemispheric), Inter-L (inter-lobe) and Intra-L (intra-lobe). The Module column denotes the functional modules that the linked two nodes belong to. The Role column denotes the roles that the two nodes play in terms of their intra- and inter-module connectivity patterns (See Materials and Methods). L: left; R: right. For the description of the abbreviations, see Table S1.

#### Edge removal

Similar to the nodal removal analysis, we also evaluated the effect of ‘lesion’ of edges on the overall topology of brain functional networks [29], [58], [59]. As expected, the continuous attacks on the global edges with high *E_bc_* had a more significant impact on the whole network integrity than the random failure of edges (Figure 9B). Further analysis indicated that the brain networks were considerably more vulnerable to the targeted attacks on inter-module connections than on intra-module connections. Particularly, we observed that the size of the largest connected network component was reduced to 20% when all inter-module connections (41/337; 12.17%) were attacked, whereas it remained nearly unchanged when the same proportion of intra-module connections were removed (Figure 9B). Analogous to the behaviors the responses to the nodal removal, the brain network also demonstrated critical points on both the global bridges (53/337; 15.73% and 80/337; 23.74%) and inter-module connections (37/337; 10.98%) attacks (Figure 9B). Together, our results indicate that the inter-module connections are accountable for a vast majority of the deleterious effects observed when the brain network is attacked.

### The Reproducibility of Our Results

**Figure 10:**
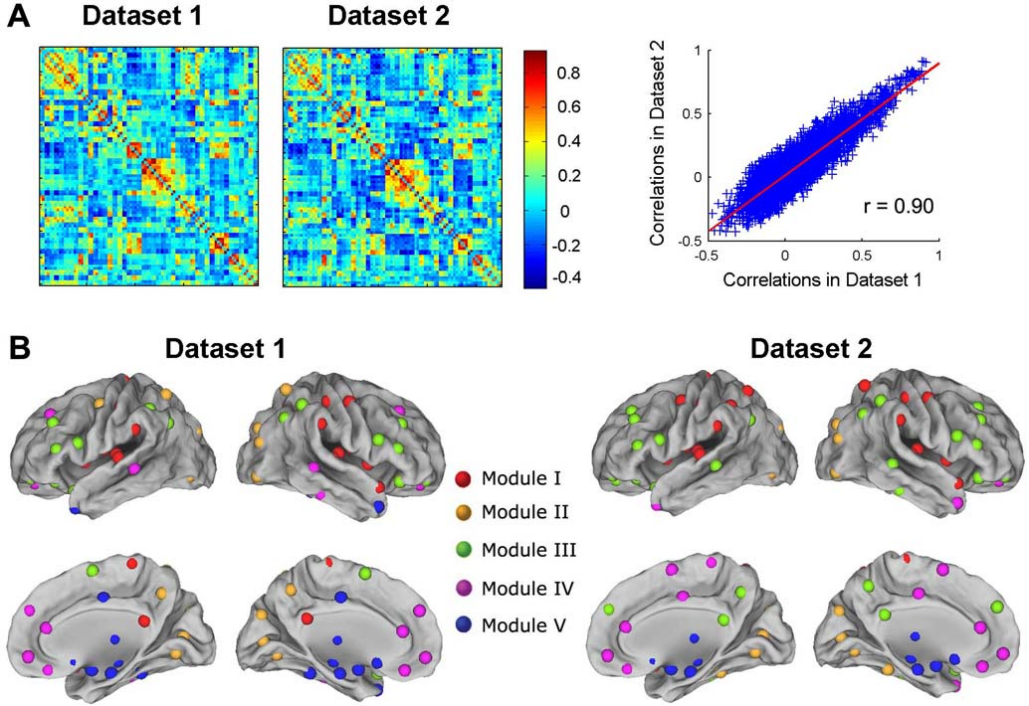
Reproducibility assessment of our results. (A) The two correlation matrices are separately shown (left: dataset 1; right: dataset 2). The right graph shows the correlation (*r* = 0.90) between dataset 1 and dataset 2. The results show that there is a high similarity in correlation patterns between dataset 1 and dataset 2. (B) The modular structures of brain functional networks are separately shown (left: dataset 1; right: dataset 2). There is also a high similarity between dataset 1 and dataset 2.

One of the key characteristics of fMRI data, is their large inter-subject variability, which may dramatically influence on the robustness of group analysis [60]. To test for robustness of the construction of brain functional networks, we divided all 18 subjects into two independent datasets (9 subjects for each dataset, age- and gender-matched), and calculated the split-half reliability. For each dataset, the brain functional networks were constructed and then analyzed with the same criterion shown in the Materials and Methods. We found that the two datasets showed a high similarity in topological organization of the brain networks: (1) visual examination indicated the correlation patterns were similar between the two datasets (Figure 10A), also similar to that in the large group (Figure 1). Further statistical analysis revealed a significant correlation (*r* = 0.90, *P* = 0.00) (Figure 10A). (2) There was the same number of modules (*N* = 5) and similar modular organization between the two datasets (Figure 10 B), also similar to those shown in the large group (Figure 2 and Figure 3). These results suggest the reliability of our findings. Of note, there were also slight differences found between the brain networks of two datasets. For example, the bilateral fusiform gyri were located in module V in the dataset 1, but in module II in the dataset 2 (Figure 10B).

## Discussion

In this study, we present a comprehensive network modularity analysis of spontaneous neuronal activity in the human brain by examining coherent spontaneous BOLD fMRI fluctuations at both the temporal and spatial scales. Our main findings are as follows: 1) that the spontaneous human brain functional networks exhibit significant modular structures that are associated with many well known brain functions, and the modular structure was highly similar at the temporal and spatial scales; 2) that the local network properties of an individual module can not be correctly depicted by using the corresponding global network properties; and 3) that the spontaneous brain functional networks contain several core regions and connections that are predominantly associated with the inter-module connectors and edges, respectively, and their lesions have critical influences on the stability and robustness of the functional brain system. Taken together, our results demonstrate an intrinsically cohesive modular architecture in the spontaneous brain functional networks, which has profound implications for our understanding of the topological mechanisms underlying the spontaneous human brain activity at a large scale.

Modularity, one of the main organizational principles of complex biological networks, has been extensively studied in recent years [33]–[36], [61]. However, it remains controversial in the brain networks, with arguments concerning the existence and emergence of the modular organization [62]. Our findings provided strong evidence in supporting the presence of the modular structure in the spontaneous human brain functional networks. The highly modularized architecture elucidated here could reflect a fundamental design principle of spontaneous brain functional networks and contribute to various aspects of intrinsically functional organization of the human brain such as the balance of functional segregation and integration while conserving wiring length [63], efficient local information processing within modules [63], [64], rapid information exchange between modules [54], and high resilience to network node or edge damages [35], [65].

We identified five intrinsically cohesive modules in the spontaneous brain functional networks that correspond to several well known subsystems such as the somatosensory/motor, auditory, attention, visual, subcortical and the “default” system (see Results for details). These uncovered functional subsystems are compatible with those found in previous spontaneous BOLD fluctuations studies using ‘seed’ functional connectivity analysis [8]–[15], hierarchical clustering [16] and ICA [17]–[20]. Recently, many studies have suggested that biological network modularity arises from natural selection pressure or evolutionary constraint for adaptation to environmental demands [66]–[68], thus leading to an interesting question that whether the brain modular architecture discovered here also reflects an evolutionary preserved pattern of brain functional organization. Previous mammalian cortical network study has demonstrated several modules corresponding to similar functional subdivisions such as the somatosensory/motor, auditory, visual and fronto-limibic systems [38]. In addition, we also noted that Vincent and colleagues [69] employed a ‘seed’ functional connectivity analysis to demonstrate the coherent spontaneous BOLD fluctuations within similar functionally connected systems in anaesthetized monkey. These results imply that the modularity of the spontaneous human brain activity is likely to correspond to an evolutionary conserved pattern of brain functional organization. However, it needs to be noted that a direct network comparison between the humans and mammalians is difficult because of the discrepancies in the data acquisition, region definition and network construction. Further computational simulation and experimental studies would be necessary to explore how the modular organization evolves in the spontaneous brain functional networks.

Our results are also consistent with the modular organization reported in several recent human brain networks studies. Chen et al. [39] demonstrated for the first time such a modular architecture (sensorimotor, auditory, visual, attention and mnemonic processing) in the human brain structural network using the inter-regional correlations of cortical thickness from structural MRI. Using the white matter tracts derived from diffusion spectrum imaging, Hagmann et al. [40] also reported a modular structure in the human cortical network that is mainly associated with the visual cortex, medial parietal cortex, bilateral frontal and temporo-parietal cortex regions. Very recently, Meunier et al. [43] demonstrated that the human brain functional networks constructed from inter-regional wavelet correlations of spontaneous neuronal activity can be decomposed into three major modules: central (putatively motor and auditory/verbal), posterior (putatively visual) and a dorsal fronto-cingulo-parietal module (putatively attention and default-mode functions). Key circuit components related to the primary brain functions (e.g. motor, auditory and visual systems) were regularly detected in all studies, thus providing evidence in supporting the notions that coherent spontaneous brain activities can be shaped by the underlying brain anatomical connectivity [70], [71]. However, we also observed significant differences in the modular architectures among these studies. For example, the ‘default’ system that has been thought to participate in internal modes of cognition [72] and the subcortical system were observed in the spontaneous brain functional networks but not in the structural brain network studies [39], [40]. The discrepancies could be attributed to the use of different neuroimaging modalities (e.g. functional, structural, and diffusion MRI), research subjects population, brain parcellation strategies or statistical thresholds during the brain network construction. Thus, it would be crucial to have additional studies to systematically investigate topological organization of human brain structural and functional networks using multi-modal neuroimaging data derived from the same participants.

Another significant finding of the present study is that the module-specific topological features in the brain functional networks can not be captured by means of the corresponding global network parameters. Most human brain networks studies have been mainly focused on the global topological properties such as the average clustering coefficients, shortest path length and network efficiency [16], [23]–[30], [32], [52]. However, the brain networks have also been shown to be highly modularized [30], [38], [40], [43], and thus raises the question as to whether the global network quantities are informative enough to depict the local topological organization of the complex brain networks. Here, we speculated that module-specific topological properties might be more predictive of spontaneous brain dynamics since brain development, aging and damages are mainly associated with functional changes in specific brain systems. Particularly, we have also demonstrated that patients with obsessive-compulsive disorder exhibit abnormal topological organization (e.g. small-world properties) in the top-down attention subnetwork rather than the whole brain system (Zhang TJ, Wang JH, Yan, CG, Gong QY, He Y; unpublished data). Therefore, our findings of the module-specific network properties could potentially have a major impact on the understanding of topological organization of complex brain networks in normal and pathological conditions.

We identified twelve network hubs that played major roles in the global coordination of the spontaneous brain activity in humans. These regions were mainly composed of recently evolved heteromodal or unimodal association cortex regions and primitive paralimbic/limbic cortex regions (See Results). The former have been shown to have rich and convergent inputs from multiple other cortical regions and contribute to the integrity of multiple functional systems, such as attention and memory systems [47]. The latter tend to be highly interconnected with the prefrontal regions and subcortical regions (e.g. nucleus accumbens) and are mainly involved in emotional processing and the maintenance of consciousness state of mind [47]. Our findings of high topological centrality in the primitive paralimbic/limibic cortex regions may also provide evidence for the ‘preferential attachment’, an important concept in the network evolution in which new nodes are preferentially attached to the nodes that are already well connected [73]. Recently, Kaiser and colleagues have argued that the emergence of network hubs could be mostly a by-product of brain evolution and development generating anatomical structures for efficient information processing [58]. We also noted that the brain functional networks prevent the presence of extremely high topological centrality as demonstrated by the truncated power-law connectivity distribution (Figure 6). It is worth mentioning that most of these global network hubs are also identified in both the functional brain networks derived from inter-regional wavelet correlations [23], [43] and structural brain networks derived from inter-regional correlations of cortical thickness [28]. However, they showed little overlap with those of structural brain networks constructed by white matter tracts from diffusion MRI [30], [32], [40]. These discrepancies could be due to the distinct brain organization information provided by different neuroimaging modalities. The topological similarities and differences in the multi-modal brain network nodes would be an important research topic in the future. Another interesting finding is that the brain regions can be divided into distinct classes in terms of their intra- and inter-module connectivity patterns. It was noted that that previously identified global hubs were predominantly associated with those network connectors linking different functional modules (referred as the party hubs in Han et al. [35]) rather than the provincial hubs occupying central positions within a single module (referred as the date hubs in Han et al. [35]). The lesions (deletions) of the network connectors also had a more deleterious effect on the network stability than the others. These findings indicated that the modular connectors were crucial for the global coordination of information flow in the brain functional networks, which was in accordance with a previous study in the mammalian brain networks showing the existence of connector hubs and their importance for maintaining network integrity [65]. In this study, network bridges are referred to those pivotal connections for the information flow of the whole brain network. We identified a set of bridges that were mainly composed of inter-module connections which ensure the communications between different functional modules. Most of these bridges are also associated with the network connectors identified previously (40/42; 95.24%), suggesting their involvements in the integrity of multiple brain functions. As expected, the lesions (deletions) of these bridges have a more negative effect on the performance of the whole brain functional networks than others.

In this study, we found a high similarity between the modular structure of temporal and spatial brain functional networks that were derived by computing temporal and spatial correlations among brain regions, respectively. As described previously, temporal correlations represent the extent of temporal coupling between two regions, but spatial correlations represent the extent of similarity of temporal correlation maps of the regions. Thus, the similar modular structure indicates that the regions within the same brain system not only have coherent spontaneous fluctuations across time, but also exhibit similar temporal connectivity patterns with the other brain regions, which could due to that the regions are associated with similar brain functions or have structural connections. Such a phenomenon has been recently observed in several functional brain systems (e.g. oculomotor and attention) in humans and monkey [15], [69].

It is important to point out that there are only three previous studies exploring the modular structure of spontaneous brain functional networks in the rats [41] and human brain [42], [43]. In contrast to the previous studies, we reported novel findings by demonstrating (1) that there was a high similarity in the modular structure of the human brain functional networks at both the temporal and spatial scales, (2) that module-specific network properties could not be represented by computing the whole-brain network characteristics, and (3) that both brain regions and connections could be classified into different categories, and their lesions exhibited different influences on brain network performance. Nonetheless, there are still some issues that need to be elucidated in future studies. First, we applied a network comparison algorithm [55] to compare the module-specific properties with the global properties of the brain networks. For each real module *s* with *n_s_* nodes, we generated the random modules by randomly selecting a set of *n_s_* nodes from the whole network. Although there was the same number of nodes between a real module and corresponding random modules, there was different edge density that likely contribute to the difference in network parameters. It would also be important to constrain the same edge density for future network comparisons. Second, in the current study, we constructed brain functional networks by computing Pearson's correlations between the time series of every pair of brain regions. Brain functional networks can also be constructed by using other connectivity measures such as the partial correlation [16], [25], wavelet correlation [23], [26] and mutual information [74]. Although the constructed brain networks by these different measures have been found to show similar network characteristics (e.g. small-world properties and modular structure), they should also have different topological organization since these connectivity measures represent different aspects (e.g. linear or nonlinear) about the relationship between brain regions. The differences in network organization and the underlying biological mechanisms associated with these connectivity measures remain to be further elucidated. Third, in the brain network construction, we removed the global brain signal to reduce the effect of physiological artifacts. However, it is still an ongoing controversy question as to the removal of the global brain signal since it could lead to ambiguous interpretations of biological mechanisms of correlations [75]. Here we also re-analyzed the modular organization of brain functional networks without the removal of global brain signals, and found that the resultant modular architecture (Figure S6) was approximately consistent with that with the removal of global signal (Figure 2 and Figure 3), suggesting a robust modular organization in spontaneous functional networks of the human brain. Fourth, we need to determine what are the mechanisms for the emergence of the modular structure and whether they are associated with the evolutionary pressure, environmental-related plasticity or genetic factors. Fifth, it is also important to investigate whether a similar modular organization exists in the brain functional networks derived from using different structural or functional subdivisions of brain regions [76]–[78] or constructed at other spatial scales such as neurons and minicolumns [71]. Finally, the functional/structural connectivity patterns of the human brain is not static, rather, it could be changed dramatically under specific task demands [9], [12], [79] or pathological conditions [25], [29], [52]. Thus, it would be very interesting to investigate how the intrinsic functional modular architecture is modulated or altered by specific tasks or brain damages, respectively.

In conclusion, we provide the empirical evidence to support the existence of the modular architecture in the spontaneous brain functional networks, thus opening a new window into our understanding of fundamental organizational principles of spontaneous neuronal activity of the human brain. Our results also suggest that the network topology-based approach provides the means to reveal potentially biological mechanism that could be responsible for brain dynamics and the underlying pathophysiology in brain disease.

## Materials and Methods

### Data Acquisition and Preprocessing

Eighteen right-handed healthy volunteers (9 male and 9 female, 21–25 years) were scanned on a 3.0 Tesla GE MR scanner (EXCITE, Milwaukee, USA). All subjects had no history of neurological or psychiatric disorders. Written informed consent was obtained from each participant and this study was approved by the Ethics Committee of Huaxi Hospital, Sichuan University. Functional images were obtained during the resting state and further preprocessed as previously described [24]. See Text S1 for details.

### Construction of Brain Functional Networks

#### Functional brain networks at the temporal scale

To construct large-scale brain functional networks at the temporal scale, a three-step process was undertaken. (i) The whole brain was first parcellated into 90 cortical and subcortical regions of interest (45 for each hemisphere, see Table S1) using a prior anatomical automatic labeling (AAL) atlas [44]. The mean time series of each region was then acquired by averaging the time series of all voxels within that region, followed by a multiple linear regression analysis to remove several sources of spurious variances from estimated head-motion profiles and global brain signal [13]. The resulting residual signal was then applied to substitute for the raw mean time series of the corresponding region. Notably, previous studies have suggested that there were significant correlations between the global brain signal and respiration-induced fMRI signal [80]. To reduce the effect of the physiological noise, we therefore removed the global brain signal as shown in several previous studies [13], [72]. (ii) We then obtained a temporal correlation matrix (90×90) for each subject by computing the Pearson correlation coefficients between the residual time series of every pair of regions as
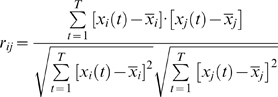
(1)where *x_i_(t)* and *x_j_(t)* (*t* = 1, 2…*T*, *T* = 190) were the residual time series of region *i* and *j* with means of 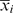 and 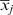, respectively. (iii) To examine whether each inter-regional correlation significantly differed from zero, two-tailed one-sample *t*-tests were performed for all the possible 4005 [i.e. (90×89)/2] pairwise correlations across subjects. Prior to the *t*-tests, a Fisher's r-to-z transform was utilized to convert each correlation coefficient *r_ij_* into *z_ij_* to improve the normality [81]. A Bonferroni-corrected significance level of *P*<0.001 was further used to threshold the correlation matrix into a binarized matrix (*S* = 8.41%. The sparisty, *S* of a network is the ratio of the number of existing edges and possible maximum edges in the network) whose element was 1 if there was significant correlation between the two brain regions and 0 otherwise. The process assured that all brain regions were included in the brain functional network and the number of false-positive connections was minimized. In this study, to evaluate whether the selection of different statistical thresholds would affect the topological stability of the brain networks, we also constructed temporal brain functional networks at several other significant *P* values of 0.005, 0.01, 0.05 and 0.10 corresponding to networks with a sparsity of 10.79%, 12.16%, 15.38% and 16.78%, respectively.

#### Functional brain networks at the spatial scale

In addition to the brain functional networks at the temporal scale, we also constructed brain functional connectivity networks at the spatial scale. First, the spatial correlation coefficient between any two brain regions [15], [69] was computed as
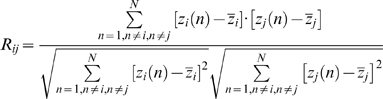
(2)where *z_i_(n)* and *z_j_(n)* (*n* = 1,2…*N*, *n*≠*i*, *n*≠*j*, *N* = 90) were the *i*th and *j*th columns of the temporal correlation matrix obtained above (after Fisher's transformation) with means of 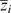 and 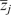, respectively. The spatial correlation coefficient between two brain regions represents the degree of similarity in the temporal functional connectivity patterns of the two regions. Second, a Fisher's r-to-z transformation was performed again and followed by a two-tail one-sample *t*-test to determine the significance of each inter-regional spatial correlation. For comparative purpose, the spatial brain functional networks were constructed based upon the thresholded spatial correlation matrix with the same sparsities as the temporal brain functional networks derived in the previous section.

### Identification of Network Modularity

The modularity *Q(p)* for a given partition *p* of the brain functional network [82] is defined as
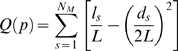
(3)where *N_M_* is the number of modules, *L* is the number of connections in the network, *l_s_* is the number of connections between nodes in module *s*, and *d_s_* is the sum of the degrees of the nodes in module *s*. The modularity index quantifies the difference between the number of intra-module links of actual network and that of random network in which connections are linked at random. The aim of this module identification process is to find a specific partition (*p*) which yields the largest network modularity, *Q(p)*. Several optimization algorithms are currently available with different advantages, here, we adopted a simulated annealing approach [45], [46] which is the most accurate to date [46], [83]. Finally, we evaluated the significance of modularity of the functional brain networks by comparing with that of 100 node- and degree-matched random networks. Notably, the partition of the network remains unchanged when multiple values of temperature were used in the simulated annealing procedure.

### Comparisons of Module-Specific and Global Network Properties

To determine whether the module-specific network properties can be captured by the global topological properties in the brain functional network, we applied a network comparison algorithm recently proposed by [55]. Briefly, for each module *s* with *n_s_* nodes, we first calculated its topological parameters, including the average degree *<k>*, clustering coefficient (*C_p_*), characteristic path length (*L_p_*), local efficiency (*E_loc_*) and global efficiency (*E_glob_*) (Text S2). We then obtained the distribution of each parameter for 1000 random modules that were generated by randomly selecting a set of *n_s_* nodes from the whole network. If the empirical module parameters fell outside of the 95% probability of the distribution, we could argue that the global network properties were unable to capture those of the local module network properties. Finally, we calculated the fraction (*r*) of modules that can not be characterized by the global network parameters.

### Characterization of Nodes and Edges

#### Node characterization

To determine the global role of each node (brain region) in the brain functional network, we computed the node betweenness centrality [56], *N^'^_bc_(i)*, as
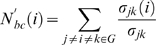
(4)where *σ_jk_* is the total number of shortest geodesic paths from node *j* to node *k*, and *σ_jk_(i)* is the number of shortest geodesic paths from node *j* to node *k* that pass through the node *i* in a graph *G*. The relative betweennness centrality, *N_bc_(i)* of a node *i* is its betweenness, *N^'^_bc_(i)* divided by the mean of all node betweenness in the network and it was calculated here by using the MatlabBGL package (http://www.stanford.edu/_dgleich/programs/matlab_bgl/). *N_bc_* measures the ability of a node over information flow between other nodes in the whole network. Regions with a high relative node betweenness centrality value (*N_bc_(i)*>mean+SD) were considered global hubs in the brain network.

To further distinguish the roles of nodes in terms of their intra- and inter-module connectivity patterns, the two measurements, the relative within-module betweenness centrality, *N_bc_^s^* and the participant coefficient, *PC* were applied [33], [34]. The *N_bc_^s^(i)* of a node *i* is the relative betweenness centrality but calculated only within the module *s* which it belongs to. It measures the importance of a node over the information flow between other nodes in the module. The *PC(i)* of a node *i* is defined as
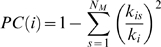
(5)where *N_M_* is the number of modules, *k_is_* is the number of links of node *i* to nodes in module *s* and *k_i_* is the total degree of node *i*. The *PC(i)* tends to 1 if node *i* has a homogeneous connection distribution with all the modules and 0 if it doesn't have any inter-module connections. *PC* measures the ability of a node in keeping the communication between its own module and the other modules. A high *PC* value for a given node usually means the node has many inter-module connections. Depending on the *N_bc_^s^*, the nodes in the brain functional network was classified into the modular hubs (*N_bc_^s^*>mean+std) and non-hubs (*N_bc_^s^*≤mean+std), respectively. In terms of the *PC*, the hub nodes were further subdivided into R1 connector hubs (*PC*>0.30) and R2 provincial hubs (*PC*≤0.30), and non-hub nodes were divided into R3 connector non-hubs (*PC*>0.30) and R4 peripheral non-hubs (*PC*≤0.30) [33], [34].

#### Edge characterization

To determine the global role of each edge (connection) in the brain functional network, we computed the edge betweenness centrality [56], *E^'^_bc_({i,j})*, as
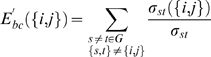
(6)where *σ_st_* is the number of shortest geodesic paths from node *s* to node *t*, and *σ_st_({i,j})* is the number of shortest geodesic paths from node *s* to node *t* that pass through the edge *(Q*. The relative betweennness centrality, *E_bc_(* [*57*] *)* of an edge *i* was its betweenness, *E^'^_bc_({i,j})* divided by the mean of all edge betweenness in the network and it was calculated here by using the MatlabBGL package (http://www.stanford.edu/_dgleich/programs/matlab_bgl/). *E_bc_({i,j})* measures the importance of an edge over information flow between other nodes in the whole network. Connections with a high relative edge betweenness centrality value (*E_bc_({i,j})*>mean+SD) were considered global bridges in the brain functional network. We further classified all edges into intra- and inter-module connections, respectively, and determined which of them were associated with the global bridges of the brain functional network.

### Network Robustness and Brain Lesions

To describe how the robustness of the brain functional network is affected by the different types of lesions, a simulated procedure was applied [23], [29], [57], [59]. Briefly, we observed the changes in both the largest connected component size and characteristic path length of the brain functional network in response to the continuous removal of the network nodes (brain regions) and edges (connections) in either a random failure or target attack fashion. As the distinct functional roles were assigned to both the network nodes and edges, target attacks on all network nodes, R1 connector hub nodes, R2 provincial hub nodes, R3 connector non-hub nodes, R4 peripheral non-hub nodes, all network connections, intra-module connections and inter-module connections were thus examined separately.

## Supporting Information

Text S1 Data Acquisition and Preprocessing
(0.01 MB PDF)

Text S2 Small-World Properties and Efficiency Measurements
(0.02 MB PDF)

Figure S1 Subdivision of Modules. (A) Subdivisions of Module I. (B) Subdivisions of Module II. (C) Subdivisions of Module III. (D) Subdivisions of Module IV. (E) Subdivisions of Module V. We applied the simulated annealing approach [38], [39] to individual modules to determine whether they can be further subdivide into small modules. The results show that there is a high modularity (Z-score>2) in four of five modules (I, II, IV and V).
(0.47 MB JPG)

Figure S2 The Modular Architectures of the Human Brain Functional Networks Constructed at Multiple Statistical Thresholds. (A) Modular structures in the functional brain networks with a sparsity of 8.41%. (B) Modular structures in the functional brain networks with a sparsity of 10.79%. (C) Modular structures in the functional brain networks with a sparsity of 12.16%. (D) Modular structures in the functional brain networks with a sparsity of 15.38%. (E) Modular structures in the functional brain networks with a sparsity of 16.78%. The first row indicates the modular structures in the temporal brain functional networks. The second row indicates the modular structures in the spatial brain functional networks. Notably, the modular structures of the temporal brain functional networks show similar patterns to those of the spatial brain functional networks. For the selection of the sparsity thresholds, see Materials and Methods.
(1.84 MB JPG)

Figure S3 The Global Hubs in the Human Brain Functional Networks (Spatial Scale). The bar plot of the occurrence that brain regions show high *Nbc* values (>mean) in the spatial brain functional networks constructed at all selected statistical thresholds (i.e. the same network sparsities as those temporal brain functional networks). The brain regions are listed according to the order of regions shown in Figure 5C. Note that the hub regions in the temporal brain functional networks (red colors) also show high topological centralities in the spatial brain functional networks. The hub regions with a high occurrence indicate that they are insensitive to the selection of statistical thresholds.
(0.27 MB JPG)

Figure S4 Topological Distribution of the Human Brain Functional Networks Constructed at Multiple Statistical Thresholds (Temporal Scale). (A) Log-log plot of the cumulative probability of node degree distribution. (B) Log-log plot of the cumulative probability of relative node betweenness distribution. (C) Log-log plot of the cumulative probability of relative edge betweenness distribution. The solid, dashed and dotted lines indicate the fits of exponentially truncated power law [*p(x)*∼x^α−1^e^x/xc^], exponential [*p(x)*∼e^x/xc^], and power law [*p(x)*∼x^α−1^], respectively. R-squared values indicate the goodness of the fits. *Retp*, R-squared value for exponentially truncated power law fit; *Re*, R-squared value for exponential fit; and *Rp*, R-squared value for power law fit. Note that these functional brain networks are constructed at the temporal scale. The spatial brain functional networks also show the similar topological distribution to the temporal brain functional networks (data not shown).
(0.23 MB JPG)

Figure S5 The Connectors of the Human Brain Functional Networks (Spatial Scale). The bar plot of the occurrence that brain regions show high *PC* values (>0.30) in the spatial functional brain networks constructed at all selected statistical thresholds (i.e. the same network sparsities as those temporal brain functional networks). The network connectors with a high occurrence indicate that they are insensitive to the selection of statistical thresholds. The brain regions are listed according to the order of regions shown in Figure 8C. Note that the connector regions in the temporal brain functional networks (red and yellow colors) also show high *PC* values in the spatial brain functional networks.
(0.34 MB JPG)

Figure S6 Surface Representation of Modular Architecture of the Human Brain Functional Network (without the removal of global brain signal). All of 90 brain regions are marked by using different colored spheres (different colors represent distinct network modules) and further mapped onto the cortical surfaces at the lateral, medial and top views, respectively, by using the Caret software [84]. The basic modular architecture (Qmax = 0.57, Z-score = 38.26) was approximately consistent with that obtained in the brain functional networks with the removal of global brain signal (Figure 3).
(0.08 MB JPG)

Table S1 Regions of Interest in the AAL-atlas.
(0.01 MB PDF)

Table S2 Modularity of the Human Brain Functional Networks. *S* indicates the network sparsity of brain functional networks that are constructed at multiple Bonferroni-corrected significance levels (*P* = 0.001, 0.005, 0.01, 0.05 and 0.10 at the temporal scale, and *P* = 0.02, 0.08, 0.14, 0.40 and 0.61 at the spatial scale, respectively) (see Materials and Methods). N_M_ denotes the number of modules in the brain functional networks and *Q* is the maximum modularity index in the modular identification (see Materials and Methods). The values in bracket indicate the mean and standard deviation values of the maximum modularity indices derived from 100 node- and degree-matched random networks.
(0.01 MB PDF)

Table S3 Global Properties of the Human Brain Functional Networks (Temporal Scale). *S* indicates the network sparsity thresholds that are used to construct temporal brain functional networks (see Materials and Methods). *N* and *K* are the number of nodes and edges in the brain networks, respectively. *<k>*, *Cp*, *Lp*,*Eloc*, and *Eglob* denote the average degree, clustering coefficient, characteristic path length, local and global efficiency, respectively. The values in bracket indicate the corresponding topological parameters derived from 100 node- and degree-matched random networks. The temporal brain functional networks were found to have a small-world structure as they had an almost identical path length (*Lpbrain/Lprandom*∼1) but were more locally clustered (*Cpbrain/Cprandom*≫1) under multiple statistical thresholds in comparison with the matched random networks.
(0.03 MB PDF)

Table S4 Global Properties of the Human Brain Functional Networks (Spatial Scale). *S* indicates the network sparsity thresholds that are used to construct spatial brain functional networks (see Materials and Methods). *N* and *K* are the number of nodes and edges in the brain networks, respectively. *<k>*, *Cp*, *Lp*, *Eloc*, and *Eglob* denote the average degree, clustering coefficient, characteristic path length, local and global efficiency, respectively. The values in bracket indicate the corresponding topological parameters derived from 100 node- and degree-matched random networks. The spatial brain functional networks were found to have a small-world structure as they had an almost identical path length (*Lpbrain/Lprandom*∼1) but were more locally clustered (*Cpbrain/Cprandom*≫1) under multiple statistical thresholds in comparison with the matched random networks.
(0.03 MB PDF)

Table S5 Module-specific and Global Brain Networks Properties (Temporal Scale). The *S* column denotes the sparsity of temporal brain functional networks. *<k>*, *Cp*, *Lp*, *Eloc*, and *Eglob* denote the average degree, clustering coefficient, characteristic path length, local and global efficiency, respectively. The values in bracket are the corresponding global network parameters that were obtained from 1,000 random modules (see Materials and Methods).
(0.01 MB PDF)

Table S6 Global vs. Module-Specific Network Properties (Spatial Scale). The table illustrates the fraction r of modules (and standard deviation) whose topological parameters significantly differ (*P*<0.05) from the corresponding global network parameters. We find all module-specific properties can not be correctly described by the global parameters because of all *r*>0.60 [47]. *<k>*, average degree; *Cp*, clustering coefficient; *Lp*, characteristic path length; *Eloc*, local efficiency; *Eglob*, global efficiency. Notably, the first column (network threshold, *S*) denotes the network sparsity thresholds that were used to construct brain functional networks at the spatial scale. Under a range of sparsity thresholds (10.79%–16.78%), there were 5 modules identified in the spatial brain functional networks, which was consistent with those of temporal brain functional networks (Table S2). Under a sparsity of threshold (8.41%), there were 6 modules found (Table S2). For details, see Materials and Methods.
(0.01 MB PDF)

Table S7 Module-specific and Global Brain Networks Properties (Spatial Scale). The *S* column denotes the sparsity of spatial brain functional networks. *<k>*, *Cp*, *Lp*, *Eloc*, and *Eglob* denote the average degree, clustering coefficient, characteristic path length, local and global efficiency, respectively. The values in bracket are the corresponding global network parameters that were obtained from 1,000 random modules (see Materials and Methods).
(0.01 MB PDF)
