# Supplementary material for: Uncovering Intrinsic Modular Organization of Spontaneous Brain Activity in Humans
Source: PLoS One. 2009 Apr 21;4(4):e5226. doi: 10.1371/journal.pone.0005226 (PMC2668183; doi:10.1371/journal.pone.0005226)
Supplement: Text S1 — Data Acquisition and Preprocessing (0.01 MB PDF) [file pone.0005226.s001.pdf]

**Text S1: Data Acquisition and Preprocessing**

*Subjects.* Eighteen right-handed healthy volunteers (9 male and 9 female, 21- 25 years) were recruited from Sichuan University. All subjects had no history of neurological or psychiatric disorders. Written informed consent was obtained from each participant and this study was approved by the Ethics Committee of Huaxi Hospital, Sichuan University.

*Data acquisitions.* All subjects were scanned on a 3.0 Tesla GE MR scanner (EXCITE, Milwaukee, USA) in Huaxi MR Research Center (HMRRC) at the West China Hospital of Sichuan University. Foam pads and headphones were used to reduce head motion and scanner noise. Functional images were obtained using an echo-planar imaging (EPI) sequence: 30 axial slices, thickness/gap = 4.5 / 0 mm, matrix = 64 × 64, repetition time = 2000 ms, echo time = 30 ms, flip angle = 90º, field of view = 220 × 220 $mm^2$. Subjects were instructed to keep their eyes closed, relax their minds and remain motionless as much as possible during the EPI data acquisition. The scan lasted for 400 s. For each subject, the first 10 volumes were discarded to allow for T1 equilibration effects and the adaptation of the subjects to the circumstances, leaving 190 volumes for further analysis.

*Data preprocessing.* Image preprocessing was carried out using the SPM5 package (http://www.fil.ion.ucl.ac.uk/spm). First, all functional images were corrected for time delay between slices and geometrical displacement due to head movement. After the correction, the images were normalized into the stereotaxic space [1], and then resampled to 3-mm isotropic voxels. Given that the coherent spontaneous BOLD fluctuations of fMRI are predominantly subtended by low-frequency components [2,3], the data were further temporally band-pass filtered (0.01 ~ 0.1 Hz) to reduce the effects of low-frequency drift and high-frequency physiological noises. The resulting data was further used to analyze the inter-regional correlations patterns.

References:

1. Talairach J, Tournoux P (1988) Co-planar stereotaxic atlas of the human brain. New York: Thieme.
2. Biswal B, Yetkin FZ, Haughton VM, Hyde JS (1995) Functional connectivity in the motor cortex of resting human brain using echo-planar MRI. Magn Reson Med 34: 537-541.
3. Lowe MJ, Mock BJ, Sorenson JA (1998) Functional connectivity in single and multislice echoplanar imaging using resting-state fluctuations. Neuroimage 7: 119-132.
