# Supplementary material for: Uncovering Intrinsic Modular Organization of Spontaneous Brain Activity in Humans
Source: PLoS One. 2009 Apr 21;4(4):e5226. doi: 10.1371/journal.pone.0005226 (PMC2668183; doi:10.1371/journal.pone.0005226)
Supplement: Text S2 — Small-World Properties and Efficiency Measurements (0.02 MB PDF) [file pone.0005226.s002.pdf]

Text S2: Small-World Properties and Efficiency Measurements

In this study, we calculated small-world parameters and efficiency measurements of the brain networks [1-3]. For a graph *G* (network) with *N* nodes and *K* edges, its average clustering coefficient, C_p and characteristic path length, L_p are computed as

$$C_p(G) = \frac{1}{N}\sum_{i\in G}\frac{2E_{G_i}}{N_{G_i}(N_{G_i}-1)},$$

$$L_p(G) = \frac{1}{N(N-1)\sum_{i\in G}\sum_{j\neq i\in G}\frac{1}{d_{ij}}}.$$

where N_{G_i} and E_{G_i} denote the number of nodes and edges within the subgraph G_i composed of neighbors of node i, and d_{ij} the minimum number of edges required to travel from node i to j. Similar to the C_p and L_p, local (E_{loc}) and global (E_{glob}) efficiency measurements of the graph G are computed as

$$E_{loc}(G) = \frac{1}{N}\sum_{i\in G}E_{loc}(i), \qquad E_{glob}(G) = \frac{1}{N}\sum_{i\in G}E_{glob}(i),$$

where $$E_{loc}(i) = \frac{1}{N_{G_i}(N_{G_i}-1)}\sum_{j\neq k\in G_i}\frac{1}{d_{jk}}, \qquad E_{glob}(i) = \frac{1}{N-1}\sum_{j\neq i\in G}\frac{1}{d_{ij}},$$

A real network would be small-world if it meets the following conditions [2,3]:

$$C_p^{real} >> C_p^{random}, L_p^{real} \approx L_p^{random} \text{ or } E_{loc}^{real} >> E_{loc}^{random}, E_{glob}^{real} \approx E_{glob}^{random}$$

where $C_p^{random}, L_p^{random}, E_{loc}^{random}, E_{glob}^{random}$ are obtained by calculating the mean values of 100 node- and degree-matched random networks [4].

References:

1. Newman MEJ (2003) The structure and function of complex networks. SIAM Review 45: 167–256.
2. Watts DJ, Strogatz SH (1998) Collective dynamics of 'small-world' networks. Nature 393: 440-442.
3. Latora V, Marchiori M (2001) Efficient behavior of small-world networks. Phys Rev Lett 87: 198701.
4. Maslov S, Sneppen K (2002) Specificity and stability in topology of protein networks. Science 296: 910-913.
