# Supplementary figures and images for: Uncovering Intrinsic Modular Organization of Spontaneous Brain Activity in Humans
Source: PLoS One. 2009 Apr 21;4(4):e5226. doi: 10.1371/journal.pone.0005226 (PMC2668183; doi:10.1371/journal.pone.0005226)

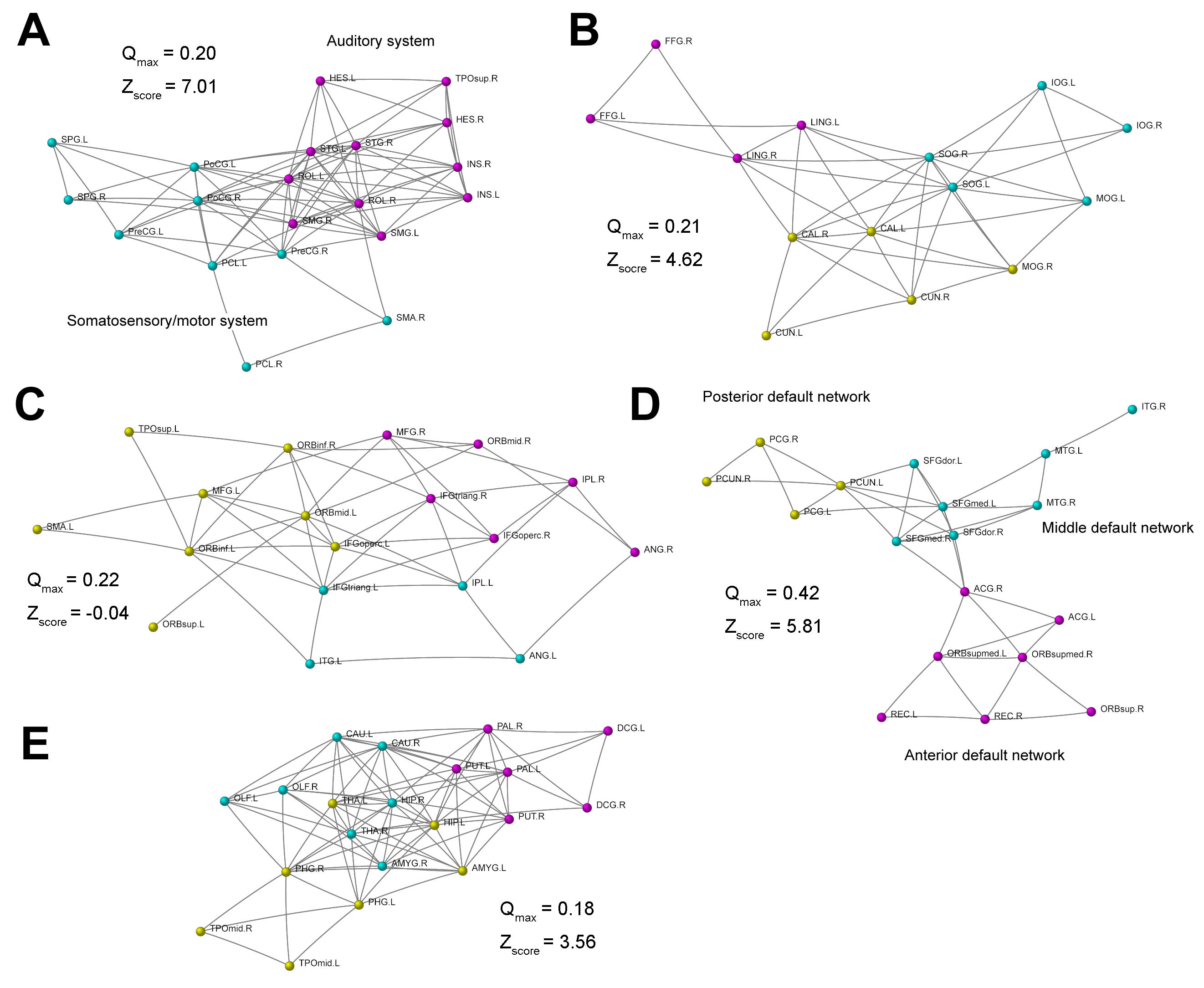

Supplement: Figure S1 — Subdivision of Modules. (A) Subdivisions of Module I. (B) Subdivisions of Module II. (C) Subdivisions of Module III. (D) Subdivisions of Module IV. (E) Subdivisions of Module V. We applied the simulated annealing approach [38], [39] to individual modules to determine whether they can be further subdivide into small modules. The results show that there is a high modularity (Z-score>2) in four of five modules (I, II, IV and V). (0.47 MB JPG) [file pone.0005226.s003.jpg]

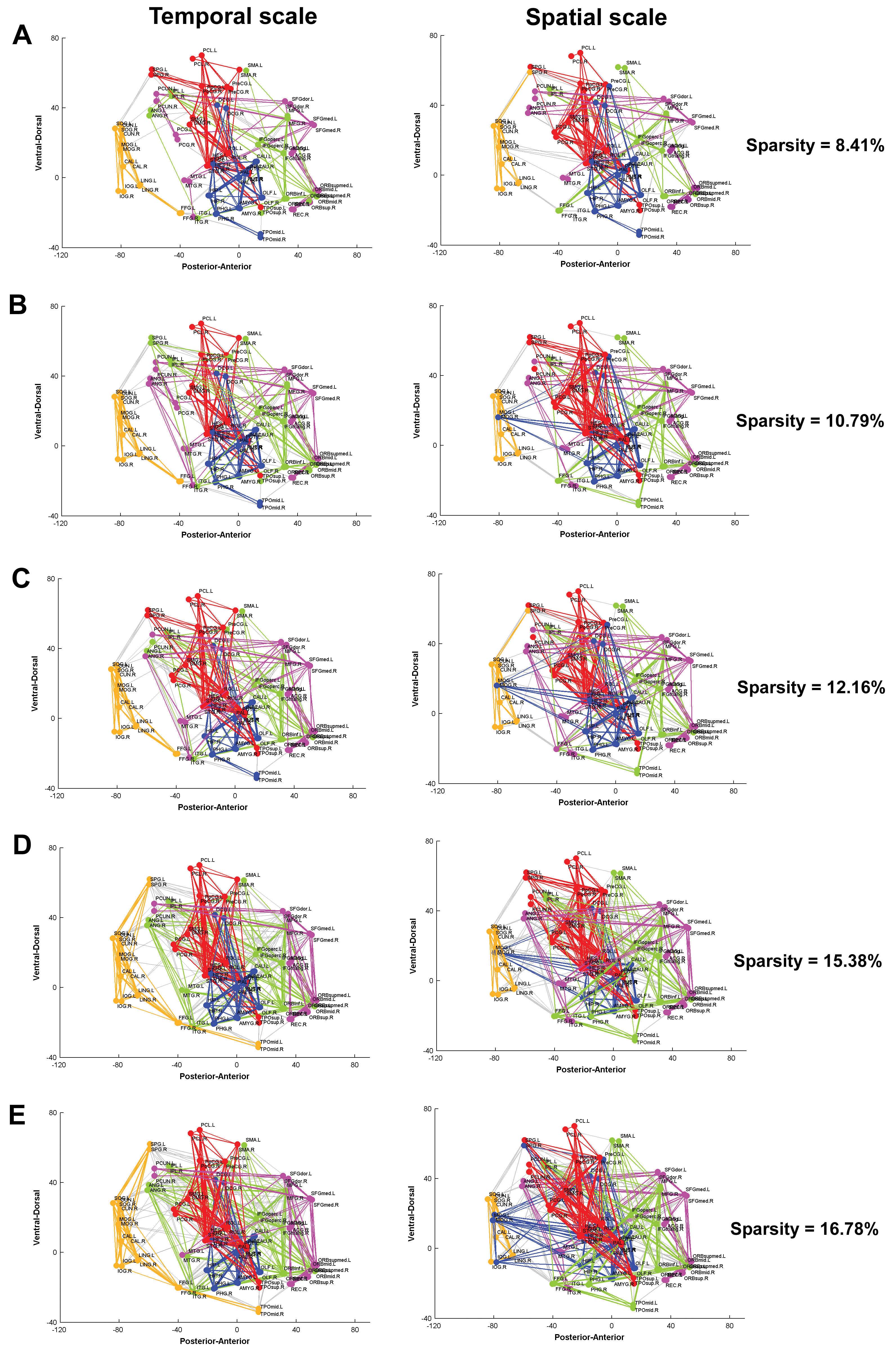

Supplement: Figure S2 — The Modular Architectures of the Human Brain Functional Networks Constructed at Multiple Statistical Thresholds. (A) Modular structures in the functional brain networks with a sparsity of 8.41%. (B) Modular structures in the functional brain networks with a sparsity of 10.79%. (C) Modular structures in the functional brain networks with a sparsity of 12.16%. (D) Modular structures in the functional brain networks with a sparsity of 15.38%. (E) Modular structures in the functional brain networks with a sparsity of 16.78%. The first row indicates the modular structures in the temporal brain functional networks. The second row indicates the modular structures in the spatial brain functional networks. Notably, the modular structures of the temporal brain functional networks show similar patterns to those of the spatial brain functional networks. For the selection of the sparsity thresholds, see Materials and Methods. (1.84 MB JPG) [file pone.0005226.s004.jpg]

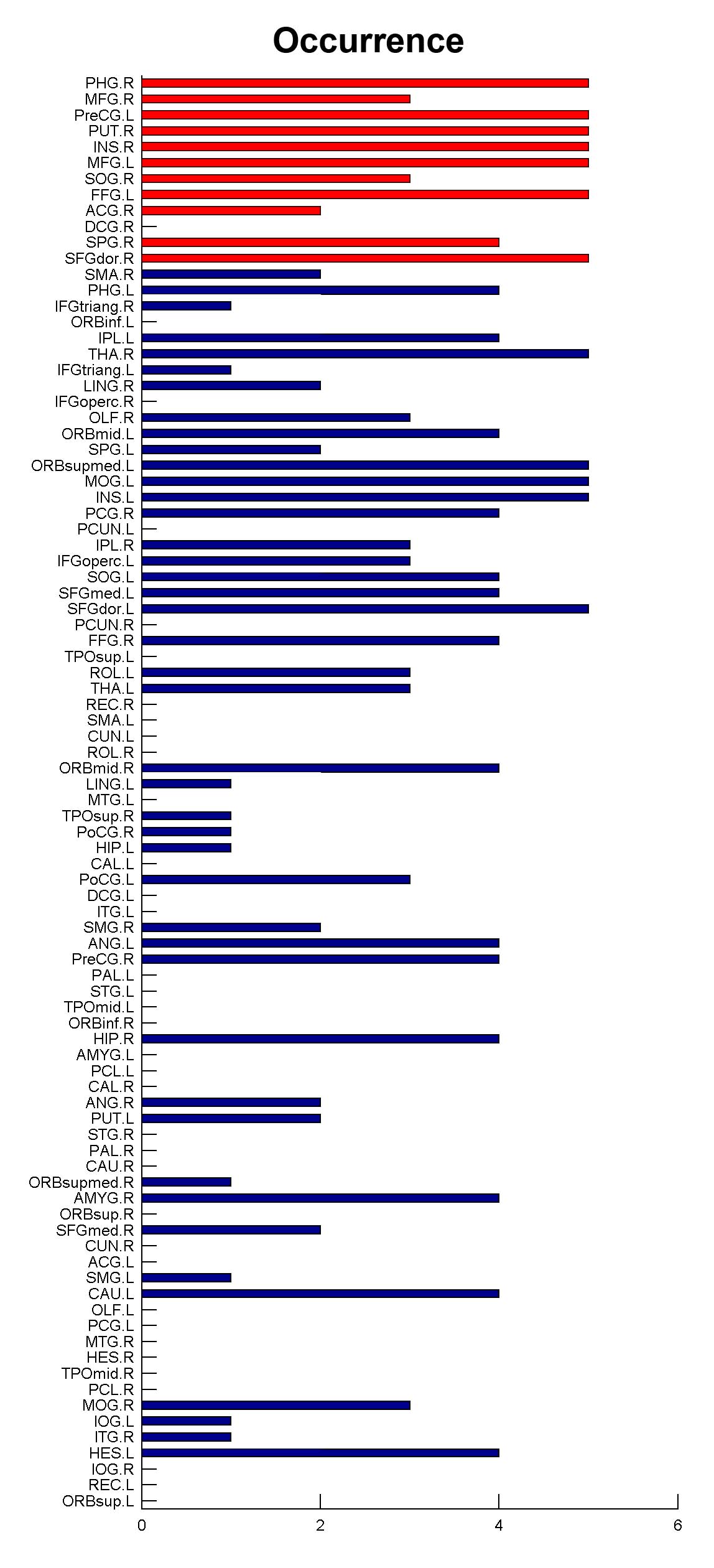

Supplement: Figure S3 — The Global Hubs in the Human Brain Functional Networks (Spatial Scale). The bar plot of the occurrence that brain regions show high Nbc values (>mean) in the spatial brain functional networks constructed at all selected statistical thresholds (i.e. the same network sparsities as those temporal brain functional networks). The brain regions are listed according to the order of regions shown in Figure 5C. Note that the hub regions in the temporal brain functional networks (red colors) also show high topological centralities in the spatial brain functional networks. The hub regions with a high occurrence indicate that they are insensitive to the selection of statistical thresholds. (0.27 MB JPG) [file pone.0005226.s005.jpg]

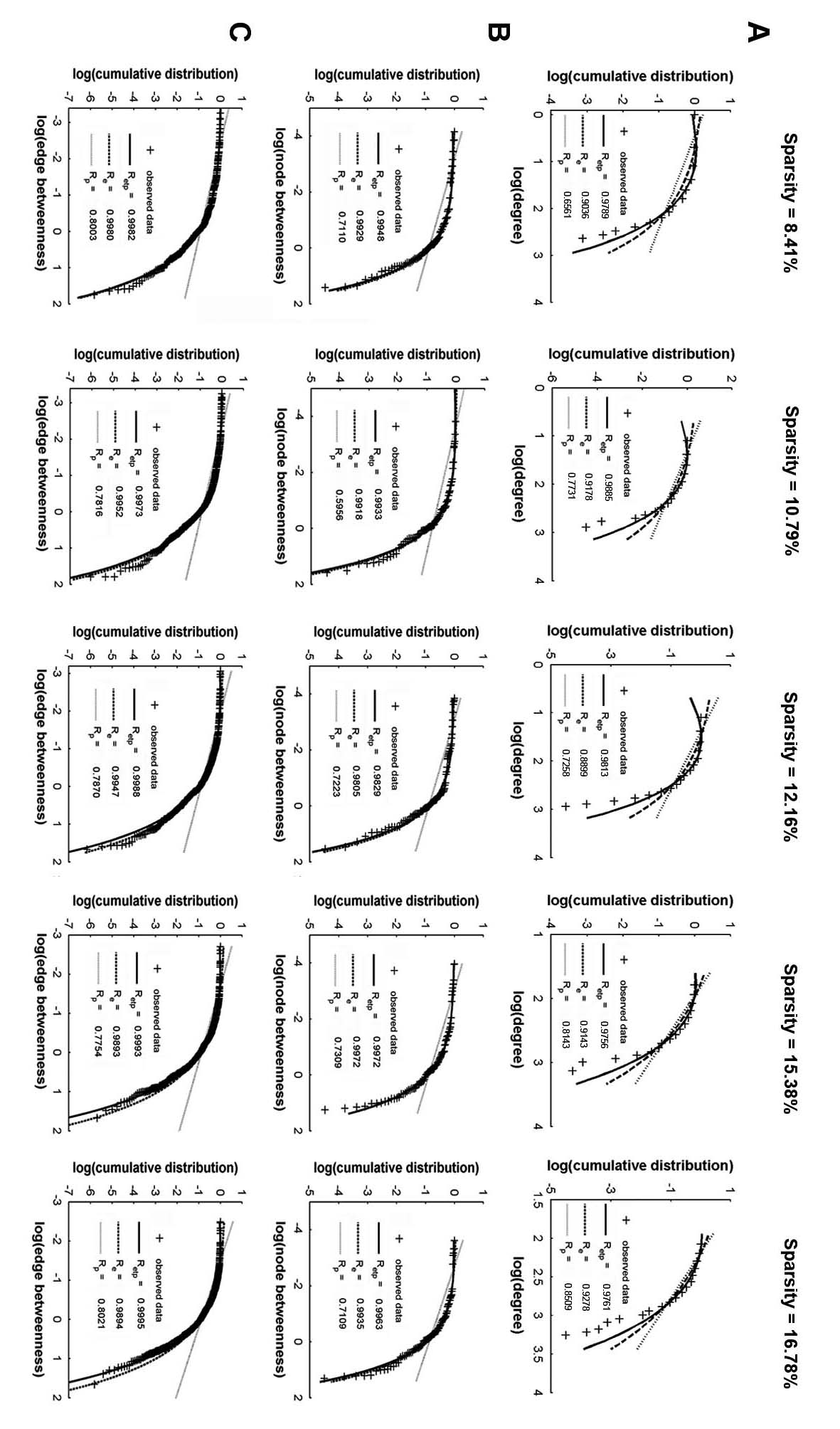

Supplement: Figure S4 — Topological Distribution of the Human Brain Functional Networks Constructed at Multiple Statistical Thresholds (Temporal Scale). (A) Log-log plot of the cumulative probability of node degree distribution. (B) Log-log plot of the cumulative probability of relative node betweenness distribution. (C) Log-log plot of the cumulative probability of relative edge betweenness distribution. The solid, dashed and dotted lines indicate the fits of exponentially truncated power law [p(x)∼xα−1ex/xc], exponential [p(x)∼ex/xc], and power law [p(x)∼xα−1], respectively. R-squared values indicate the goodness of the fits. Retp, R-squared value for exponentially truncated power law fit; Re, R-squared value for exponential fit; and Rp, R-squared value for power law fit. Note that these functional brain networks are constructed at the temporal scale. The spatial brain functional networks also show the similar topological distribution to the temporal brain functional networks (data not shown). (0.23 MB JPG) [file pone.0005226.s006.jpg]

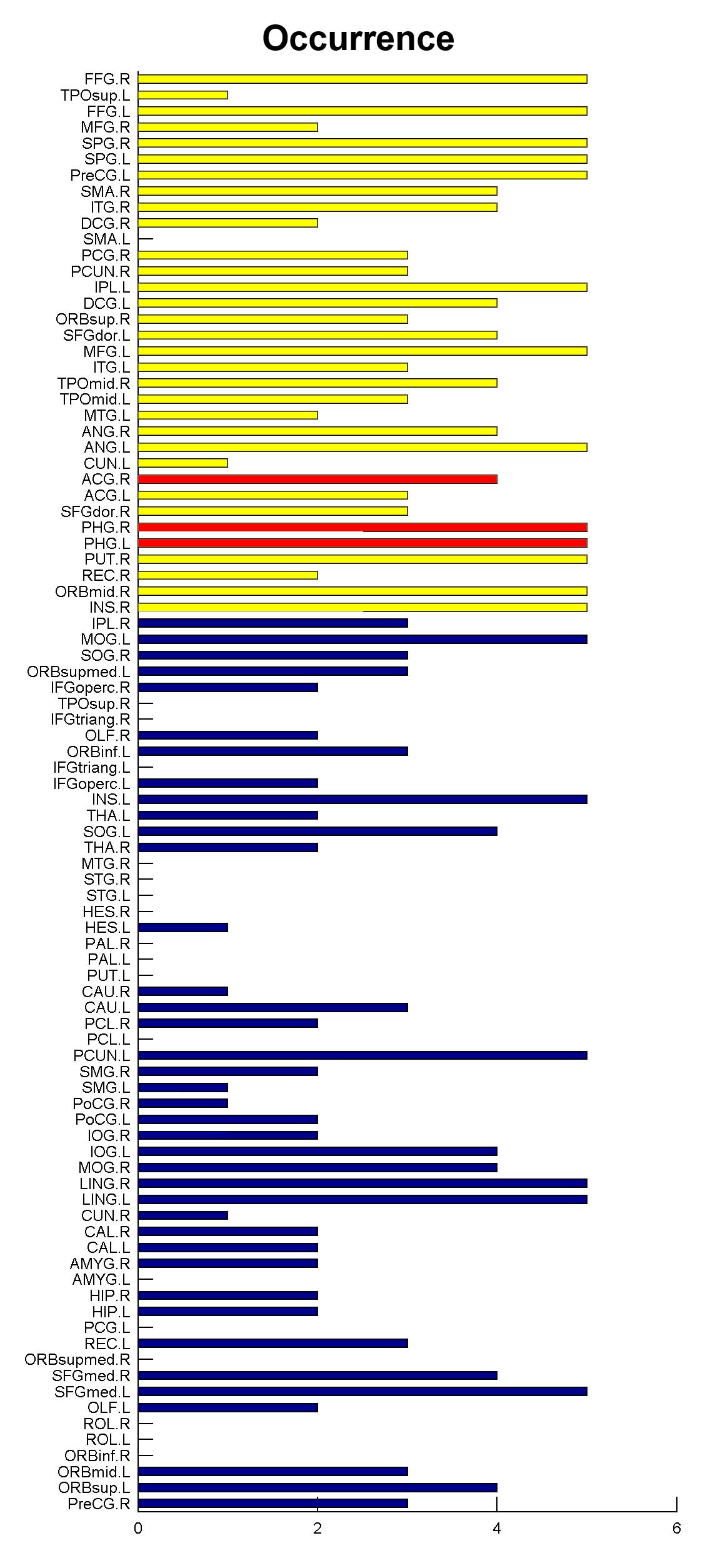

Supplement: Figure S5 — The Connectors of the Human Brain Functional Networks (Spatial Scale). The bar plot of the occurrence that brain regions show high PC values (>0.30) in the spatial functional brain networks constructed at all selected statistical thresholds (i.e. the same network sparsities as those temporal brain functional networks). The network connectors with a high occurrence indicate that they are insensitive to the selection of statistical thresholds. The brain regions are listed according to the order of regions shown in Figure 8C. Note that the connector regions in the temporal brain functional networks (red and yellow colors) also show high PC values in the spatial brain functional networks. (0.34 MB JPG) [file pone.0005226.s007.jpg]

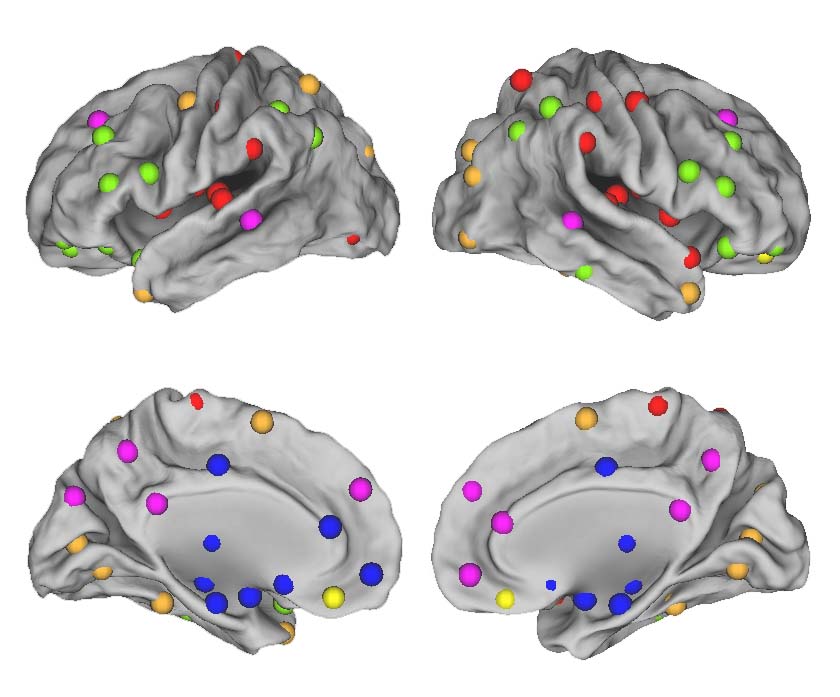

Supplement: Figure S6 — Surface Representation of Modular Architecture of the Human Brain Functional Network (without the removal of global brain signal). All of 90 brain regions are marked by using different colored spheres (different colors represent distinct network modules) and further mapped onto the cortical surfaces at the lateral, medial and top views, respectively, by using the Caret software [84]. The basic modular architecture (Qmax = 0.57, Z-score = 38.26) was approximately consistent with that obtained in the brain functional networks with the removal of global brain signal (Figure 3). (0.08 MB JPG) [file pone.0005226.s008.jpg]
