# Supplementary material for: Uncovering Intrinsic Modular Organization of Spontaneous Brain Activity in Humans
Source: PLoS One. 2009 Apr 21;4(4):e5226. doi: 10.1371/journal.pone.0005226 (PMC2668183; doi:10.1371/journal.pone.0005226)
Supplement: Table S1 — Regions of Interest in the AAL-atlas. (0.01 MB PDF) [file pone.0005226.s009.pdf]

Table S1. Regions of Interest in the AAL-atlas

Regions	Abbreviations	Regions	Abbreviations
Precentral gyrus	PreCG	Lingual gyrus	LING
Superior frontal gyrus (dorsolateral)	SFGdor	Superior occipital gyrus	SOG
Superior frontal gyrus (orbital part)	ORBsup	Middle occipital gyrus	MOG
Middle frontal gyrus	MFG	Inferior occipital gyrus	IOG
Middle frontal gyrus (orbital part)	ORBmid	Fusiform gyrus	FFG
Inferior frontal gyrus (opercular part)	IFGoperc	Postcentral gyrus	PoCG
Inferior frontal gyrus (triangular part)	IFGtriang	Superior parietal gyrus	SPG
Inferior frontal gyrus (orbital part)	ORBinf	Inferior parietal, but supramarginal and angular gyri	IPL
Rolandic operculum	ROL	Supramarginal gyrus	SMG
Supplementary motor area	SMA	Angular gyrus	ANG
Olfactory cortex	OLF	Precuneus	PCUN
Superior frontal gyrus (medial)	SFGmed	Paracentral lobule	PCL
Superior frontal gyrus (medial orbital)	ORBsupmed	Caudate nucleus	CAU
Rectus gyrus	REC	Lenticular nucleus, putamen	PUT
Insula	INS	Lenticular nucleus, pallidum	PAL
Anterior cingulate and paracingulate gyri	ACG	Thalamus	THA
Median cingulate and paracingulate gyri	DCG	Heschl gyrus	HES
Posterior cingulate gyrus	PCG	Superior temporal gyrus	STG
Hippocampus	HIP	Temporal pole: superior temporal gyrus	TPOsup
Parahippocampal gyrus	PHG	Middle temporal gyrus	MTG
Amygdala	AMYG	Temporal pole: middle temporal gyrus	TPOmid
Calcarine fissure and surrounding cortex	CAL	Inferior temporal gyrus	ITG
Cuneus	CUN		
