# Supplementary material for: Uncovering Intrinsic Modular Organization of Spontaneous Brain Activity in Humans
Source: PLoS One. 2009 Apr 21;4(4):e5226. doi: 10.1371/journal.pone.0005226 (PMC2668183; doi:10.1371/journal.pone.0005226)
Supplement: Table S2 — Modularity of the Human Brain Functional Networks. S indicates the network sparsity of brain functional networks that are constructed at multiple Bonferroni-corrected significance levels (P = 0.001, 0.005, 0.01, 0.05 and 0.10 at the temporal scale, and P = 0.02, 0.08, 0.14, 0.40 and 0.61 at the spatial scale, respectively) (see Materials and Methods). NM denotes the number of modules in the brain functional networks and Q is the maximum modularity index in the modular identification (see Materials and Methods). The values in bracket indicate the mean and standard deviation values of the maximum modularity indices derived from 100 node- and degree-matched random networks. (0.01 MB PDF) [file pone.0005226.s010.pdf]

Table S2. Modularity of the Human Brain Functional Networks

**Scale**	**Threshold, *S***	N_M	***Q***
Temporal	8.41%	5	0.66 (0.33±0.01)
	10.79%	5	0.61 (0.28±0.01)
	12.16%	5	0.58 (0.26±0.01)
	15.38%	5	0.52 (0.23±0.01)
	16.78%	5	0.50 (0.21±0.01)
Spatial	8.41%	6	0.63 (0.31±0.01)
	10.79%	5	0.59 (0.27±0.01)
	12.16%	5	0.56 (0.25±0.01)
	15.38%	5	0.49 (0.22±0.00)
	16.78%	5	0.47 (0.21±0.00)

S indicates the network sparsity of brain functional networks that are constructed at multiple Bonferroni-corrected significance levels (*P* = 0.001, 0.005, 0.01, 0.05 and 0.10 at the temporal scale, and *P* = 0.02, 0.08, 0.14, 0.40 and 0.61 at the spatial scale, respectively) (see Materials and Methods). N_M denotes the number of modules in the brain functional networks and *Q* is the maximum modularity index in the modular identification (see Materials and Methods). The values in bracket indicate the mean and standard deviation values of the maximum modularity indices derived from 100 node- and degree-matched random networks.
