# Supplementary material for: Uncovering Intrinsic Modular Organization of Spontaneous Brain Activity in Humans
Source: PLoS One. 2009 Apr 21;4(4):e5226. doi: 10.1371/journal.pone.0005226 (PMC2668183; doi:10.1371/journal.pone.0005226)
Supplement: Table S3 — Global Properties of the Human Brain Functional Networks (Temporal Scale). S indicates the network sparsity thresholds that are used to construct temporal brain functional networks (see Materials and Methods). N and K are the number of nodes and edges in the brain networks, respectively. , Cp, Lp,Eloc, and Eglob denote the average degree, clustering coefficient, characteristic path length, local and global efficiency, respectively. The values in bracket indicate the corresponding topological parameters derived from 100 node- and degree-matched random networks. The temporal brain functional networks were found to have a small-world structure as they had an almost identical path length (Lpbrain/Lprandom∼1) but were more locally clustered (Cpbrain/Cprandom≫1) under multiple statistical thresholds in comparison with the matched random networks. (0.03 MB PDF) [file pone.0005226.s011.pdf]

Table S3. Global Properties of the Human Brain Functional Networks (Temporal Scale)

**Threshold, *S***	***N***	***K***	**<*k*>**	C_p	L_p	E_{loc}	E_{glob}
8.41%	90	337	7.49	0.48 (0.10±0.01)	2.70 (2.19±0.01)	0.62 (0.13±0.02)	0.37 (0.46±0.00)
10.79%	90	432	9.60	0.53 (0.12±0.01)	2.34 (2.00±0.00)	0.69 (0.18±0.01)	0.43 (0.50±0.00)
12.16%	90	487	10.82	0.53 (0.13±0.01)	2.20 (1.92±0.00)	0.71 (0.23±0.02)	0.45 (0.52±0.00)
15.38%	90	616	13.69	0.54 (0.16±0.01)	1.98 (1.79±0.00)	0.74 (0.35±0.02)	0.50 (0.56±0.00)
16.78%	90	672	14.93	0.54 (0.17±0.01)	1.91 (1.75±0.00)	0.74 (0.40±0.01)	0.52 (0.57±0.00)

S indicates the network sparsity thresholds that are used to construct temporal brain functional networks (see Materials and Methods). *N* and *K* are the number of nodes and edges in the brain networks, respectively. <*k*>, C_p, L_p, E_{loc} and E_{glob} denote the average degree, clustering coefficient, characteristic path length, local and global efficiency, respectively. The values in bracket indicate the corresponding topological parameters derived from 100 node- and degree-matched random networks. The temporal brain functional networks were found to have a small-world structure as they had an almost identical path length ($L_p^{brain}/L_p^{random} \sim 1$) but were more locally clustered ($C_p^{brain}/C_p^{random} >> 1$) under multiple statistical thresholds in comparison with the matched random networks.
