# Supplementary material for: Uncovering Intrinsic Modular Organization of Spontaneous Brain Activity in Humans
Source: PLoS One. 2009 Apr 21;4(4):e5226. doi: 10.1371/journal.pone.0005226 (PMC2668183; doi:10.1371/journal.pone.0005226)
Supplement: Table S4 — Global Properties of the Human Brain Functional Networks (Spatial Scale). S indicates the network sparsity thresholds that are used to construct spatial brain functional networks (see Materials and Methods). N and K are the number of nodes and edges in the brain networks, respectively. , Cp, Lp, Eloc, and Eglob denote the average degree, clustering coefficient, characteristic path length, local and global efficiency, respectively. The values in bracket indicate the corresponding topological parameters derived from 100 node- and degree-matched random networks. The spatial brain functional networks were found to have a small-world structure as they had an almost identical path length (Lpbrain/Lprandom∼1) but were more locally clustered (Cpbrain/Cprandom≫1) under multiple statistical thresholds in comparison with the matched random networks. (0.03 MB PDF) [file pone.0005226.s012.pdf]

Table S4. Global Properties of the Human Brain Functional Networks (Spatial Scale)

**Threshold, *S***	***N***	***K***	**<*k*>**	C_p	L_p	E_{loc}	E_{glob}
8.41%	90	337	7.49	0.50 (0.11±0.01)	2.86 (2.28±0.02)	0.63 (0.17±0.02)	0.35 (0.44±0.00)
10.79%	90	432	9.60	0.55 (0.14±0.01)	2.38 (2.02±0.01)	0.70 (0.24±0.02)	0.42 (0.49±0.00)
12.16%	90	487	10.82	0.57 (0.16±0.01)	2.21 (1.94±0.01)	0.73 (0.29±0.02)	0.45 (0.52±0.00)
15.38%	90	616	13.69	0.55 (0.19±0.01)	1.97 (1.81±0.00)	0.74 (0.40±0.02)	0.51 (0.55±0.00)
16.78%	90	672	14.93	0.53 (0.20±0.01)	1.90 (1.76±0.00)	0.73 (0.44±0.01)	0.53 (0.57±0.00)

S indicates the network sparsity thresholds that are used to construct spatial brain functional networks (see Materials and Methods). *N* and *K* are the number of nodes and edges in the brain networks, respectively. <*k*>, C_p, L_p, E_{loc} and E_{glob} denote the average degree, clustering coefficient, characteristic path length, local and global efficiency, respectively. The values in bracket indicate the corresponding topological parameters derived from 100 node- and degree-matched random networks. The spatial brain functional networks were found to have a small-world structure as they had an almost identical path length ($L_p^{brain}/L_p^{random} \sim 1$) but were more locally clustered ($C_p^{brain}/C_p^{random} >> 1$) under multiple statistical thresholds in comparison with the matched random networks.
