# Supplementary material for: Uncovering Intrinsic Modular Organization of Spontaneous Brain Activity in Humans
Source: PLoS One. 2009 Apr 21;4(4):e5226. doi: 10.1371/journal.pone.0005226 (PMC2668183; doi:10.1371/journal.pone.0005226)
Supplement: Table S6 — Global vs. Module-Specific Network Properties (Spatial Scale). The table illustrates the fraction r of modules (and standard deviation) whose topological parameters significantly differ (P<0.05) from the corresponding global network parameters. We find all module-specific properties can not be correctly described by the global parameters because of all r>0.60 [47]. , average degree; Cp, clustering coefficient; Lp, characteristic path length; Eloc, local efficiency; Eglob, global efficiency. Notably, the first column (network threshold, S) denotes the network sparsity thresholds that were used to construct brain functional networks at the spatial scale. Under a range of sparsity thresholds (10.79%–16.78%), there were 5 modules identified in the spatial brain functional networks, which was consistent with those of temporal brain functional networks (Table S2). Under a sparsity of threshold (8.41%), there were 6 modules found (Table S2). For details, see Materials and Methods. (0.01 MB PDF) [file pone.0005226.s014.pdf]

Table S6. Global vs. Module-Specific Network Properties (Spatial Scale)

**Threshold, *S***	**<*k*>**	C_p	L_p	E_{loc}	E_{glob}
8.41%	1.00 (0.00)	0.83 (0.00)	1.00 (0.00)	1.00 (0.02)	1.00 (0.00)
10.79%	1.00 (0.00)	0.86 (0.09)	1.00 (0.00)	1.00 (0.00)	1.00 (0.00)
12.16%	1.00 (0.00)	1.00 (0.00)	1.00 (0.00)	1.00 (0.00)	1.00 (0.00)
15.38%	1.00 (0.00)	1.00 (0.00)	1.00 (0.00)	1.00 (0.00)	1.00 (0.00)
16.78%	1.00 (0.00)	1.00 (0.00)	1.00 (0.00)	1.00 (0.00)	1.00 (0.00)

The table illustrates the fraction *r* of modules (and standard deviation) whose topological parameters significantly differ ($P < 0.05$) from the corresponding global network parameters. We find all module-specific properties can not be correctly described by the global parameters because of all $r > 0.60$ [47]. <*k*>, average degree; C_p, clustering coefficient; L_p, characteristic path length; E_{loc}, local efficiency; E_{glob}, global efficiency. Notably, the first column (network threshold, *S*) denotes the network sparsity thresholds that were used to construct brain functional networks at the spatial scale. Under a range of sparsity thresholds (10.79%-16.78%), there were 5 modules identified in the spatial brain functional networks, which was consistent with those of temporal brain functional networks (Table S2). Under a sparsity of threshold (8.41%), there were 6 modules found (Table S2). For details, see Materials and Methods.
