# Supplementary material for: Uncovering Intrinsic Modular Organization of Spontaneous Brain Activity in Humans
Source: PLoS One. 2009 Apr 21;4(4):e5226. doi: 10.1371/journal.pone.0005226 (PMC2668183; doi:10.1371/journal.pone.0005226)
Supplement: Table S7 — Module-specific and Global Brain Networks Properties (Spatial Scale). The S column denotes the sparsity of spatial brain functional networks. , Cp, Lp, Eloc, and Eglob denote the average degree, clustering coefficient, characteristic path length, local and global efficiency, respectively. The values in bracket are the corresponding global network parameters that were obtained from 1,000 random modules (see Materials and Methods). (0.01 MB PDF) [file pone.0005226.s015.pdf]

Table S7. Module-specific and Global Brain Networks Properties (Spatial Scale)

**Threshold, *S***	**Module**	$\langle k \rangle$	C_p	L_p	E_{loc}	E_{glob}
8.41%	M I	10.10 (1.60±0.46)	0.70 (0.26±0.13)	1.35 (9.20±4.10)	0.78 (0.21±0.14)	0.74 (0.13±0.05)
	M II	4.77 (1.05±0.47)	0.71 (0.17±0.16)	1.49 (12.76±9.72)	0.85 (0.17±0.17)	0.67 (0.11±0.06)
	M III	5.25 (1.26±0.45)	0.55 (0.20±0.14)	1.56 (10.68±5.49)	0.70 (0.21±0.15)	0.64 (0.12±0.05)
	M IV	2.50 (0.58±0.39)	0.35 (0.07±0.15)	1.62 (14.26±8.81)	0.41 (0.07±0.15)	0.62 (0.10±0.07)
	M V	3.80 (0.77±0.45)	0.41 (0.10±0.15)	1.47 (14.86±11.19)	0.46 (0.11±0.16)	0.68 (0.10±0.07)
	M VI	8.48 (1.67±0.46)	0.71 (0.27±0.13)	1.46 (8.92±3.74)	0.85 (0.29±0.13)	0.68 (0.13±0.05)
10.79%	M I	11.10 (2.29±0.53)	0.75 (0.35±0.12)	1.28 (5.53±2.13)	0.86 (0.38±0.13)	0.78 (0.21±0.07)
	M II	11.00 (1.08±0.50)	0.78 (0.17±0.18)	1.34 (9.97±7.48)	0.86 (0.18±0.18)	0.74 (0.14±0.08)
	M III	5.45 (1.85±0.53)	0.75 (0.30±0.13)	1.30 (6.78±3.01)	0.87 (0.32±0.14)	0.77 (0.18±0.07)
	M IV	6.33 (1.92±0.55)	0.60 (0.31±0.14)	1.49 (6.62±2.96)	0.75 (0.33±0.15)	0.67 (0.18±0.07)
	M V	4.32 (2.04±0.52)	0.53 (0.33±0.13)	1.96 (6.22±2.61)	0.62 (0.35±0.14)	0.51 (0.19±0.07)
12.16%	M I	11.52 (2.46±0.55)	0.82 (0.38±0.12)	1.28 (4.69±1.76)	0.91 (0.41±0.13)	0.78 (0.24±0.08)
	M II	6.00 (1.35±0.50)	0.74 (0.22±0.18)	1.29 (7.54±4.83)	0.87 (0.23±0.18)	0.77 (0.17±0.08)
	M III	7.06 (1.95±0.53)	0.63 (0.32±0.14)	1.40 (5.76±2.69)	0.76 (0.34±0.15)	0.71 (0.21±0.08)
	M IV	5.00 (2.28±0.54)	0.63 (0.36±0.12)	1.81 (5.09±2.04)	0.76 (0.39±0.14)	0.55 (0.22±0.08)
	M V	12.10 (2.30±0.55)	0.80 (0.35±0.13)	1.23 (4.97±2.00)	0.90 (0.38±0.14)	0.81 (0.23±0.08)
15.38%	M I	7.45 (3.53±0.62)	0.80 (0.45±0.10)	1.15 (2.78±0.69)	0.90 (0.50±0.11)	0.87 (0.38±0.07)
	M II	9.26 (1.60±0.56)	0.66 (0.25±0.18)	1.32 (5.39±3.40)	0.83 (0.27±0.19)	0.76 (0.24±0.11)
	M III	5.78 (2.78±0.60)	0.66 (0.40±0.12)	1.60 (3.38±1.31)	0.80 (0.44±0.14)	0.62 (0.33±0.09)
	M IV	12.56 (2.61±0.59)	0.84 (0.38±0.13)	1.15 (3.56±1.36)	0.92 (0.42±0.15)	0.87 (0.31±0.09)
	M V	12.50 (2.63±0.59)	0.79 (0.39±0.13)	1.31 (3.52±1.31)	0.89 (0.42±0.14)	0.77 (0.32±0.09)
16.78%	M I	13.52 (3.55±0.66)	0.81 (0.45±0.10)	1.20 (2.60±0.60)	0.91 (0.50±0.11)	0.84 (0.40±0.07)
	M II	7.11 (1.34±0.54)	0.94 (0.19±0.19)	1.06 (5.89±4.47)	0.97 (0.20±0.20)	0.94 (0.24±0.12)
	M III	6.11 (3.17±0.63)	0.68 (0.42±0.11)	1.57 (2.80±0.83)	0.81 (0.47±0.12)	0.64 (0.38±0.08)
	M IV	12.82 (2.84±0.61)	0.81 (0.40±0.13)	1.24 (3.09±1.07)	0.91 (0.44±0.14)	0.80 (0.35±0.09)
	M V	7.11 (3.32±0.63)	0.94 (0.43±0.11)	1.06 (2.74±0.72)	0.97 (0.48±0.12)	0.94 (0.38±0.08)

The *S* column denotes the sparsity of spatial brain functional networks. $\langle k \rangle$, C_p, L_p, E_{loc} and E_{glob} denote the average degree, clustering coefficient, characteristic path length, local and global efficiency, respectively. The values in bracket are the corresponding global network parameters that were obtained from 1000 random modules (see Materials and Methods).
